# Degradation of Perfluoroalkyl Acids under 222 nm Irradiation in the Presence and Absence of Sulfite

**DOI:** 10.1021/acsestengg.6c00161

**Published:** 2026-05-02

**Authors:** Bahngmi Jung, Garrett McKay

**Affiliations:** Zachry Department of Civil & Environmental Engineering, Texas A&M University, College Station, Texas 77845, United States

**Keywords:** PFAS, PFBS, advanced reduction processes, hydrated electron, krypton chloride excimer lamp

## Abstract

Krypton chloride excimer (KrCl*) lamps emitting at 222 nm have recently been shown to enhance the fluence-normalized rates of micropollutant abatement in UV-oxidation and -reduction systems relative to conventional, 254 nm light sources. Here, we investigated the destruction of short- and long-chain perfluoroalkyl acids under 222 nm irradiation from KrCl* lamps in the presence and absence of sulfite. At pH 12 under anaerobic conditions, all compounds were degraded under 222 nm irradiation with apparent quantum yields (Φ_app,PFAS_) increasing in the order 0.036 (perfluorobutanesulfonate (PFBS)), 0.124 (perfluorooctanesulfonate (PFOS)), 0.374 (perfluorooctanoic acid (PFOA)), and 0.822 (perfluorobutanoic acid (PFBA)), which are markedly higher than quantum yields reported previously at pH 8.5. Multiple lines of evidence suggest that the observed UV_222_ only degradation of perfluorocarboxylic acids is due to both direct photolysis and e_aq_^–^-based destruction, the latter enabled by photolysis of hydroxide ions at pH 12. In contrast, PFBS and PFOS degradation in the UV_222_ only system is due to e_aq_^–^-mediated destruction, not true direct photolysis. To compare the effectiveness of 222 nm KrCl* lamps with 254 nm, the UV/sulfite system was tested on two complex water matrices: (i) a reverse osmosis concentrate (ROC) spiked with short- and long-chain perfluoroalkyl acids and (ii) an aqueous film forming foam (AFFF). The extent of PFAS destruction in these complex matrices was greater for 222 nm irradiation relative to 254 nm by up to 6-fold when normalized to incident fluence. Overall, this study provides fundamental insight into the UV-based degradation of perfluoroalkyl acids at 222 nm and suggests that KrCl* lamps may provide improved performance compared to conventional 254 nm sources in UV/sulfite ARP for PFAS destruction in complex water matrices.

## Graphical abstract

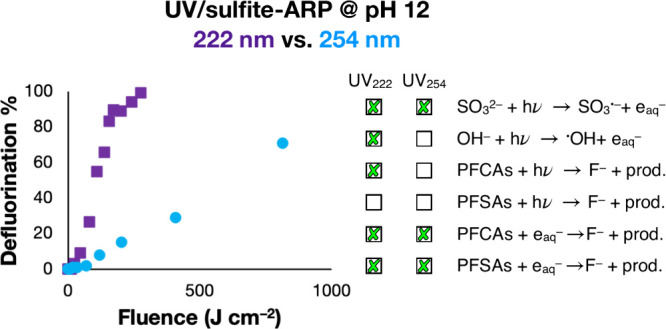

## 1 Introduction

This study explores 222 nm-emitting krypton chloride (KrCl*) excimer lamps as a light source for the destruction of per- and polyfluoroalkyl substances (PFAS) in ultraviolet-based advanced reduction processes (UV-ARP), which have shown promise for the reductive transformation of PFAS via reactions with hydrated electrons (e_aq_^–^).^1–7^ KrCl* lamps have been an area of recent interest in water treatment,^8–10^ including the direct photolysis of micropollutants,^11^ indirect photolysis via oxidizing radicals,^12–17^ and reductive degradation of oxyanions and PFAS.^18–20^ Collectively, these studies have demonstrated enhanced fluence-normalized degradation rates for 222 nm irradiation compared to 254 nm. Although mechanistic work is still ongoing, the reasons for the enhancement are likely a combination of enhanced molar absorptivities and quantum yields at 222 nm and radical chemistry initiated by water matrix components (e.g., dissolved oxygen^21^).

Several recent reports have also tested the ability of KrCl* lamps to degrade PFAS.^18–20^ At circumneutral pH, quantum yields for direct photolysis (Φ_*PFAS*_) were reported to be significant for perfluorocarboxylic acids (PFCAs) (Φ_*PFAS*_ up to ∼10%) while sulfonic acids had negligible photoreactivity (Φ_*PFAS*_ ≈ 0).^18^ The rate constant for perfluorooctanoic acid (PFOA) direct photolysis was not significantly impacted by pH (from 5.0 to 10.5) nor the presence of dissolved oxygen, bicarbonate, or phosphate.^18^ However, compared to bicarbonate buffered solution, the rate of PFOA degradation was lowered 4-fold in wastewater containing nitrate (11 mg L^–1^) and dissolved organic matter (6 mg_C_ L^–1^), two water matrix constituents known to both shield light and scavenge hydrated electrons (e_aq_^–^).^4^ In the UV/sulfite-advanced reduction process (ARP), two studies have demonstrated larger fluence-normalized rate constants of multiple PFAS at 222 nm vs 254 nm in ultrapure water.^19,20^ This was attributed to the higher molar absorptivity of sulfite at 222 nm, resulting in a greater fluence-normalized electron exposure (*R* *_e^–^_,_UV_*) measured by chloroacetate.^19,20^

Based on these studies, KrCl* lamps offer a promising approach for enhancing rates of PFAS destruction via UV-ARP. However, there are several knowledge gaps that need to be explored. First, the UV/sulfite system under 254 nm irradiation maximizes PFAS destruction at basic pH,^4,22^ but prior 222 nm direct photolysis and UV-ARP studies focus on circumneutral pH. Second, prior studies have not compared the performance of 222 vs 254 nm treatment in the presence of water matrix components. Although Xin et al. demonstrated that synthetic solutions of nitrate, dissolved organic matter (DOM), or bicarbonate decrease the rate of PFOS destruction in the UV/sulfite ARP at 222 nm,^19^ the combined effect of these species (e.g., in a real water matrix) was not assessed. Water matrix components have different photochemical activities at 222 and 254 nm, suggesting that their impact on UV-ARP performance will differ between the two wavelengths. Waste streams concentrated in PFAS (e.g., ion exchange regenerant brines or “still bottoms”) are also concentrated in water matrix components; thus, understanding their role on the efficacy of UV-ARP destruction of PFAS at 222 nm is critical.

This contribution focuses on the degradation of short- and long-chain perfluoroalkyl acids under UV-only and UV-ARP conditions. Most experiments were conducted at pH 12, which was previously shown to enhance UV/sulfite-ARP destruction of perfluoroalkyl acids at 254 nm.^22^ Apparent quantum yields under 222 nm irradiation for PFAS destruction (Φ_app, PFAS_) and quantum yields of fluoride release (Φ_app, *F* ^–^_) were calculated at pH 12, which were compared to previously reported values at pH 8.5.^18^ We investigated the mechanism of UV-only degradation by evaluating the impact of solution pH, dissolved oxygen, and methanol addition on the degradation of individual PFAS and the e_aq_^–^ probe compound monochloroacetic acid (MCAA).^5^ To compare the efficacy of UV_222_ to conventional UV_254_-ARP, we tested PFAS defluorination in two complex matrices. The first was a reverse osmosis concentrate (ROC) containing high levels of light-shielding and e_aq_^–^-scavenging water matrix components. The second was an aqueous film forming foam (AFFF), previously characterized by ^19^F-NMR and studied in the UV_254_/sulfite ARP.^23^ Taken together, this study advances the potential application of KrCl* lamps for UV-ARP treatment of PFAS by exploring the mechanism of PFAS destruction under UV_222_ only conditions at pH 12 and presenting fluence-normalized comparisons of the UV_222_- and UV_254_/sulfite-ARP in complex water matrices.

## 2 Materials and Methods

### 2.1 Chemicals and Solutions

The chemicals used in this study are provided in the Supporting Information (Text S1, Table S1). PFAS standards and surrogates were obtained from Wellington Laboratories (Table S2). The preparation of PFAS stock solutions and standards is described in Text S7. All stock solutions were made by dissolving the chemicals in ultrapure water (18.2 MΩ-cm) obtained from a Barnstead Water Purification System (ThermoFisher) and stored at 4 °C. ROC was collected from the Groundwater Replenishment System of the Orange County Water District (OCWD) (see SI Text S2 and Table S3).

### 2.2 Photochemical Experiments

Photodegradation experiments utilized filtered KrCl* lamps (USHIO) emitting light at 222 nm (UV_222_), focused 4 cm above the surface of a sealed quartz cell (Starna) with a measured path length of 1.02 cm (see Figure S1 and Text S10). The measured temperature in the quartz cells at equilibrium was 20.5 °C. All experimental solutions were deoxygenated by purging with nitrogen gas (industrial grade, Airgas) for 30–120 min, unless otherwise specified. The incident UV fluence rate (*E*_0_) received in the reaction solution was measured using the iodide-iodate actinometry, yielding values of *E*_0_ = 2.1 to 3.68 × 10^‑9^ mol photons cm^–2^ s^–1^ (Text S3). *E*_0_ measured by iodide-iodate actinometry was used to compare 222 and 254 nm systems (see Table S4),^5,24^ consistent with prior literature.^14^ The UV fluence (mJ cm^–2^) reported in this study was calculated as the product of the incident fluence rate (mJ cm^–2^ s^–1^) and exposure time (s).

Fluoride ion (F^–^) concentrations were measured using an ion selective electrode and were validated through comparison with ion chromatography (Text S4, Figure S2). The defluorination percentage (deF%) is defined as the ratio of [F^–^] released into solution to the total concentration of C–F bonds. For individual PFAS, the concentration of C–F bonds was based on the initial PFAS concentration and molecular formula, whereas for AFFF it was based on the total organic fluorine measurement via ^19^F-NMR by Tenorio et al.^23^ Fluence-based deF% (% cm^2^ J^–1^) is defined as the defluorination percentage normalized to the incident fluence at the corresponding reaction time. Concentrations of sulfite and other anions present in ROC were measured during photochemical experiments. Additional details are provided in Texts S5 and S6.

All PFAS measurements were performed with a triple quadrupole mass spectrometer (Altis, ThermoFisher Scientific, Waltham, MA) coupled to a binary pump HPLC (Vanquish, ThermoFisher Scientific). Additional details are provided in Text S7.

**Figure 1:**
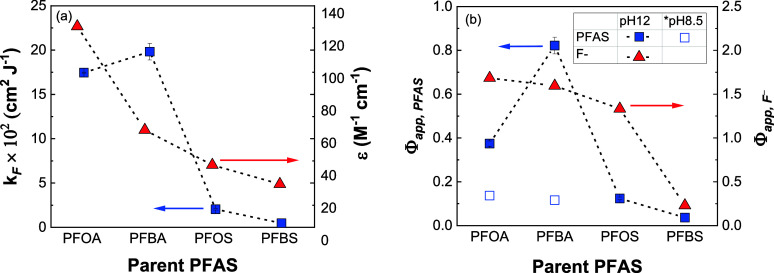
Fluence-based rate constants (k_*F*_) and molar absorptivity (ε) (a), apparent quantum yield for PFAS decay (Φ_app, PFAS_) and fluoride ion formation (Φ_app, *F* ^–^_) (b) at pH 12 under UV_222_ irradiation. Conditions: [PFBA]_0_ = [PFOA]_0_ = [PFBS]_0_ = [PFOS]_0_ = 25 μM, pH = 12, N_2_-purged, and incident UV fluence rate = 2.8 × 10^–9^ mol photons cm^–2^ s^–1^. Markers represent the mean of duplicate measurements, and error bars indicate the range between duplicates. Φ_app, PFAS_ and Φ_app, *F* ^–^_ are based on eqs 1 and 2, respectively. *Φ_PFAS_ at pH 8.5 are from Xin et al.^18^

## 3 Results and Discussion

### 3.1 UV_222_ Photolysis

To evaluate the importance of UV only conditions on PFAS degradation in the UV_222_/sulfite-ARP, single compound solutions of 25 μM PFBA, PFOA, PFBS, and PFOS were irradiated at 222 nm, followed by parent compound and fluoride ion quantification. The PFAS decay and defluorination profiles are shown in Figure S4. First-order photodegradation rate constants for the decay of PFOA, PFBA, PFOS, and PFBS at pH 12 in N_2_-purged solution were quantified based on time and incident fluence and are shown in Figure 1 (see Text S8 and Table S10). Φ_app, PFAS_ and Φ_*app*, *F* ^–^_ values at 222 nm under pH 12 in N_2_-purged solution were calculated via eqs 1 and 2, respectively:

$$\Phi_{\mathrm{app},\mathrm{PFAS}}=\frac{k_{\mathrm{obs},\mathrm{PFAS}}[\mathrm{PFAS}{]}_{0}}{\frac{E_{0}}{l}(1-10^{-(\varepsilon_{\mathrm{PFAS}}[\mathrm{PFAS}{]}_{0}+\varepsilon_{{\mathrm{OH}}^{-}}[{\mathrm{OH}}^{-}])l})\times \left(\frac{\varepsilon_{\mathrm{PFAS}}[\mathrm{PFAS}{]}_{0}}{\varepsilon_{\mathrm{PFAS}}[\mathrm{PFAS}{]}_{0}+\varepsilon_{{\mathrm{OH}}^{-}}[{\mathrm{OH}}^{-}]}\right)}$$ 1

$$\Phi_{\mathrm{app},F^{-}}=\frac{R_{0}^{F^{-}}}{\frac{E_{0}}{l}(1-10^{-(\varepsilon_{\mathrm{PFAS}}[\mathrm{PFAS}{]}_{0}+\varepsilon_{{\mathrm{OH}}^{-}}[{\mathrm{OH}}^{-}])l})\times \left(\frac{\varepsilon_{\mathrm{PFAS}}[\mathrm{PFAS}{]}_{0}}{\varepsilon_{\mathrm{PFAS}}[\mathrm{PFAS}{]}_{0}+\varepsilon_{{\mathrm{OH}}^{-}}[{\mathrm{OH}}^{-}]}\right)}$$ 2

where *k*_obs,PFAS_ is the PFAS first-order photodegradation rate constant (s^–1^), [PFAS]_0_ and [OH^–^] are the initial PFAS and OH^–^ concentration (mol L^–1^), *E*_0_ is the incident fluence rate at 222 nm (mmol photons cm^–2^ s^–1^), *l* is the effective path length (cm), and *ε*_PFAS_ and *ε*_OH^–^_ the molar absorptivity of the PFAS and OH^–^ at 222 nm (M^–1^ cm^–1^), and *R*_0_^F^–^^ is the initial rate of fluoride ion formation (M s^–1^). Additional details are provided in SI Text S10. To account for the contribution of indirect pathways under high pH conditions, the term “apparent quantum yield” was used to distinguish it from the quantum yield with direct photolysis at neutral pH under 222 nm (see Section 3.3).

The fluence-based first-order rate constants measured at pH 12 followed the order: PFBS (4.67 × 10^–3^ cm^2^ J^–1^) < PFOS (2.04 × 10^–2^ cm^2^ J^–1^) < PFOA (0.175 cm^2^ J^–1^) < PFBA (0.198 cm^2^ J^–1^). Similarly, Φ_app, PFAS_ at pH 12 increased in the order PFBS (0.036) < PFOS (0.124) < PFOA (0.374) < PFBA (0.822) (Figure 1). The Φ_app, PFAS_ measured for PFBA and PFOA at pH 12 are larger than those reported at pH 8.5^18^ by 7.1 and 2.7-fold, respectively. Notably, PFOS photolysis at 222 nm was reported by Xin et al. to be negligible at pH 8.5,^18^ but we observed appreciable degradation of both PFOS and PFBS at pH 12. At pH 8.5, Xin et al. showed that molar absorptivity was positively correlated with the photolysis rate constant for PFCAs and GenX but not other PFAS classes (e.g., PFSAs).^18^ In our study, where photolysis was performed at pH 12, PFCAs had a higher molar absorptivity and also a shorter half-life (*t*_1/2_ ∼ 40 min) and achieved complete defluorination earlier than PFSAs (*t*_1/2_ = 6.2–27 h), which had lower molar absorptivities at pH 12 (Table S15). All PFAS studied are highly acidic, with reported p*K*_a_ values < 3 (Table S15); thus, the observed differences in degradation rate at pH 8.5 and 12 cannot be explained by PFAS speciation. Notably, near 100% defluorination of PFBS was observed after 48 h of 222 nm irradiation at pH 12 (Figure S4), albeit at a slower rate than PFCAs, not reported in prior KrCl* studies. Φ_app, *F* ^–^_ for PFOA, PFBA, and PFOS exceeding unity is consistent with existing *e*_aq_^–^-based degradation mechanisms in that multiple fluoride ions are lost in the decarboxylation-hydroxylation-elimination-hydrolysis pathway.^6^

### 3.2 Effect of pH and Dissolved Oxygen on UV_222_ Only Treatment

Φ_*PFAS*_ values for PFOA (0.137) and PFBA (0.116) at pH 8.5^18^ are lower than those reported here at pH 12 of 0.374 ± 0.012 and 0.822 ± 0.038, respectively. To investigate the origin of these differences, we measured parent compound degradation kinetics, defluorination, and production of intermediates during photolysis of PFBA, PFOA, PFBS, and PFOS in the presence of dissolved oxygen at pH 12 and at pH 9 under N_2_ saturation (Figure 2).

#### 3.2.1 Perfluorocarboxylic Acids

Under N_2_-purged conditions, rate constants for loss of PFCAs during UV_222_ treatment were greater at pH 12 than at pH 9 (see Figures 2a,b and SI Table S10). For example, fluence-normalized rate constants for PFOA and PFBA at pH 9 were 0.139 and 0.059 cm^2^ J^–1^, respectively, compared to 0.175 and 0.198 cm^2^ J^–1^ at pH 12 (Figure 2c). Although Xin et al. reported no difference in the extent of PFOA degradation after 4 h of UV_222_ between pH 5 and 10.5,^18^ examination of their data show a slight increase in the initial rate of PFOA loss with increasing pH. The larger impact of pH observed for PFBA (3.4-fold) compared to PFOA (1.3-fold) suggests that the pH-dependence for UV_222_ only degradation may be chain length-dependent. In addition, our experiments conducted at an initial pH of 9 and 12 did not use buffer, whereas Xin et al. buffered solutions with 5 mM bicarbonate.^18^ The pH 12 system remained stable over the course of irradiation (pH 12 ± 0.2), whereas solutions irradiated initially at pH 9 dropped rapidly to pH ∼ 4 (PFCAs) or pH ∼ 5 (PFSAs) (see Figure S12). At pH 9, the rapid pH drop may be due to HF elimination during early stages of photolysis.

The presence of dissolved oxygen substantially decreased the degradation rate of PFOA and PFBA at pH 12 compared to the N_2_-purged system. For example, fluence-normalized rate constants for PFOA and PFBA at pH 12 with dissolved oxygen were 0.115 and 0.055 cm^2^ J^–1^, respectively, compared to 0.175 and 0.198 cm^2^ J^–1^ with N_2_ purging (Figure 2 and Table S10). This decrease observed at pH 12 contrasts with the lack of dissolved oxygen impact on PFOA photolysis at 222 nm at pH 8.5 reported by Xin et al.^18^ Comparing the effects of pH and N_2_ purging indicates that the degradation of PFOA and PFBA were more inhibited by the presence of dissolved oxygen, with reductions of 51.9% and 79.6%, respectively, than by the decrease in pH (41.5% for PFOA and 69.4% for PFBA) (Table S10). In contrast, the fluoride formation rate at *t* = 4 h (*R* ^*F* ^–^^) (Table S10) and overall deF% were more suppressed by the decrease in pH from 12 to 9 than by the presence of dissolved oxygen (Figure 2). Specifically, the presence of dissolved oxygen reduced *R* ^*F* ^–^^ by 54.8 and 56.3% for PFOA and PFBA, respectively, whereas lowering pH caused larger reductions of 69.4 and 90.5%. These results indicate that the degradation of parent PFCAs by UV_222_ is more adversely affected by the presence of dissolved oxygen, while the extent of defluorination is more sensitive to pH reduction.

**Figure 2:**
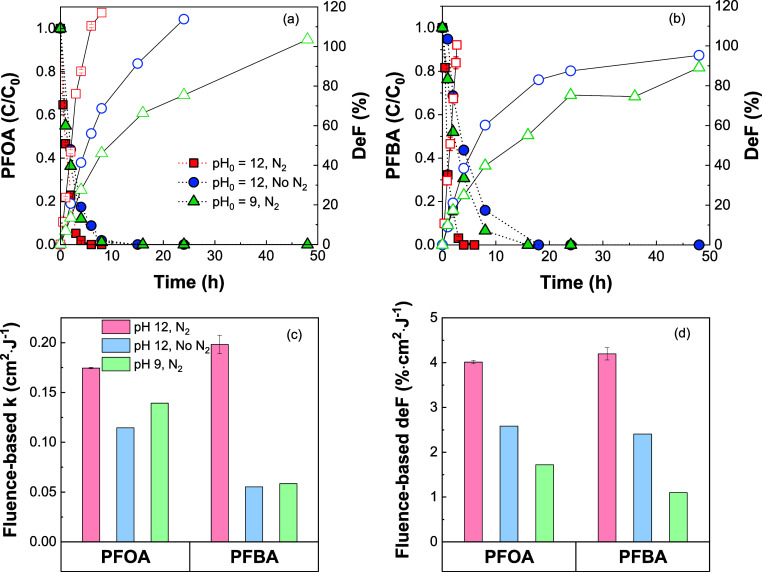
Comparison of parent compound decay kinetics and defluorination of PFOA and PFBA at varying pH and in the presence of dissolved oxygen. Degradation and defluorination time courses of (a) PFOA and (b) PFBA. Open symbols represent deF% and closed symbols represent normalized parent compound concentration. (c) Fluence-normalized rate constants and (d) defluorination percentages for PFCAs at pH 9 and 12 and in the presence of dissolved oxygen. Conditions: [PFBA]_0_ = [PFOA]_0_ = 25 μM, incident UV fluence rate = 2.1–3.0 × 10^–9^ mol photons cm^–2^ s^–1^ and the solution pH was adjusted to 12 or 9 using 5 N NaOH (no buffer). More detailed conditions are provided in Table S4.

The formation and persistence of shorter chain intermediates were also influenced by the presence of dissolved oxygen and solution pH. Under N_2_-purged conditions at pH 12, degradation products including perfluoroheptanoic acid (PFHpA), perfluorohexanoic acid (PFHxA), and PFBA were observed during photolysis of PFOA and were degraded within ∼4 h (Figure S8). Perfluoropropanoic acid (PFPrA) was observed during photolysis of PFBA (Figure S9). Experiments in the presence of dissolved oxygen at pH 12 showed a larger molar yield of intermediate products relative to N_2_-purged solutions at both pH 9 and pH 12. PFOA photolysis demonstrated a clear time-dependence in the production of shorter-chain perfluorocarboxylic acids (Figures S8 and S9). Under UV_222_ only conditions, the observed reactivity and defluorination of PFCAs were influenced by dissolved oxygen and high pH. These findings suggest that degradation proceeds through a combination of direct photolysis and indirect mechanisms involving reactive species generated from hydroxide (see Section 3.3).

#### 3.2.2 Perfluorosulfonic Acids

In contrast to PFCAs, PFOS and PFBS were not appreciably degraded (<10% defluorination and loss of parent compound) at pH 9 under N_2_ purging nor at pH 12 in the presence of dissolved oxygen (Figures S10 and S11). The lack of UV_222_ degradation of PFOS and PFBS at pH 9 is consistent with the negligible Φ_PFAS_ for PFSAs reported by Xin et al. at pH 8.5.^18^ At pH 12 in the absence of dissolved oxygen, near 100% defluorination was reached for both PFOS (at 24 h) and PFBS (at 48 h) (Figure S10). The lack of PFSA degradation at lower pH and in the presence of dissolved oxygen suggests that a mechanism other than direct photolysis is responsible for the loss of these compounds.

Sulfate production was observed during UV_222_ only treatment of PFOS and PFBS, and its temporal pattern closely matched that of fluoride (Figure 3). Specifically, negligible sulfate or fluoride was produced during the first 3 h of PFOS photolysis (<5% desulfonation or defluorination) (Figure 3a), with production rapidly increasing between 3 and 4 h. This lag period was also observed for the degradation and defluorination kinetics of PFBS (Figure 3b) but was absent for both PFOA and PFBA (Figure S4).

**Figure 3:**
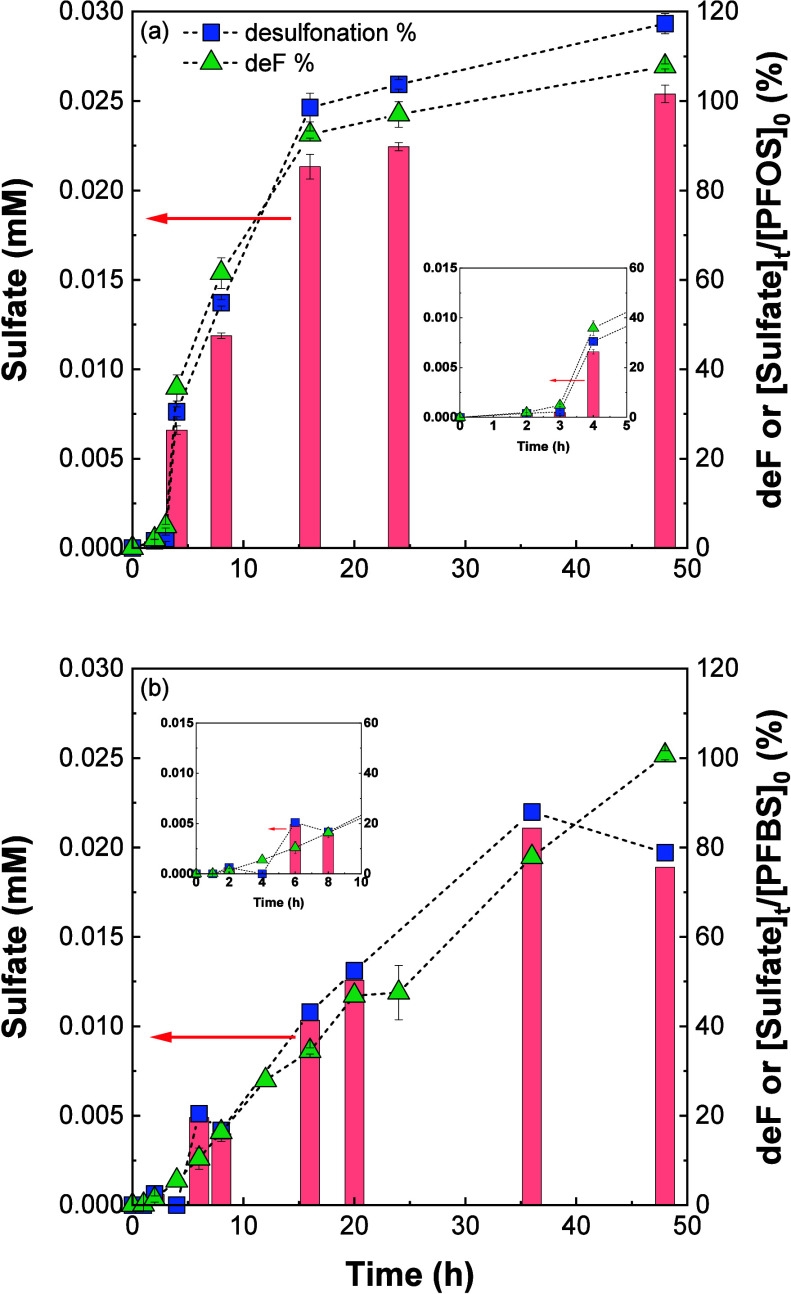
Desulfonation and defluorination of (a) PFOS and (b) PFBS under UV_222_ photolysis. Conditions: [PFOS]_0_ = [PFBS]_0_ = 25 μM, N_2_ purged, in the absence of sulfite, pH_0_ = 12, and incident UV fluence rate = 2.8 × 10^–9^ mol photons cm^–2^ s^–1^.

### 3.3 Mechanism of UV_222_ Degradation at pH 12

Based on experiments performed at pH 8.5, Xin et al. proposed photodecarboxylation as the initial step in the degradation of PFCAs under 222 nm irradiation.^18^ In our study, we observed photolytic degradation of PFSAs only at pH 12 and under N_2_ purging, whereas PFCAs were degraded at circumneutral pH and in the presence of dissolved oxygen, albeit at a lower rate. These observations, the time profiles of shorter-chain intermediates during PFOA photolysis, and the kinetics of defluorination and desulfonation of PFSAs form the basis for the proposed mechanism.

We hypothesize that 222 nm-mediated degradation of PFOS and PFBS at pH 12 and N_2_ purged conditions in the absence of sulfite is mediated by e_aq_^–^ produced by photolysis of the hydroxide ion (eq 3).^25^

OH−+hν→eaq−+OH• 3

Although OH^–^ does not typically absorb at 222 nm, we observed significant red-shifting of the UV absorbance spectrum at pH > 11, a phenomenon reported previously,^26^ such that at pH 12 and greater, the overlap between KrCl* and hydroxide spectra is significant (Figure S27). A quantum yield of e_aq_^–^ formation by OH^–^ photolysis by 193 nm laser excitation has been measured by Sauer et al. as ∼0.1,^27^ but no values to our knowledge are available at 222 nm.

In the presence of dissolved oxygen, e_aq_^–^ is scavenged by O_2_ forming superoxide radical anion (eq 4)^28^

$$\begin{array}{cc} O_{2}+{e_{\mathrm{aq}}}^{-}\to {O_{2}}^{•-} & k=1.9\times 10^{10}\;M^{-1}\;s^{-1} \end{array}$$ 4

At pH 12, ^•^OH undergoes deprotonation to form O^•–^ (eq 5)^29^

OH•⇌O•−+H+pKa=11.5 5

Reactions of O_2_^•–^ with ^•^OH and O^•–^ regenerate dissolved oxygen and hydroxide (eqs 6–8)^30–32^

O2•−+OH•→O2+OH−k=1×1010M−1s−1 6

$$\begin{array}{cc} {O_{2}}^{•-}+O^{•-}\to O_{2}+2{\mathrm{OH}}^{-} & k=6.0\times 10^{8}\;M^{-1}\;s^{-1} \end{array}$$ 7

$$\begin{array}{cc} O^{•-}+{e_{\mathrm{aq}}}^{-}\to {\mathrm{OH}}^{-} & k=2.2\times 10^{10}\;M^{-1\;}s^{-1} \end{array}$$ 8

The regeneration of dissolved oxygen via eqs 6 and 7 will lead to continuous scavenging of e_aq_^–^, explaining the lack of PFBS and PFOS degradation observed at pH 12 in the presence of dissolved oxygen. Under N_2_ purged conditions, the low concentration of dissolved oxygen is anticipated to slow the rate of eq 4 thereby decreasing the rate of eqs 6 and 7, allowing sufficient e_aq_^–^ to accumulate for PFAS degradation once O_2_ is consumed. The lag period observed in Figure 3 is consistent with trace dissolved oxygen removal impeding PFAS destruction at early stages of the reaction. Because absorption of light by hydroxide is hypothesized to be the primary photolytic pathway, the Φ_app, PFAS_ in Figure 1 was recalculated using the rate of light absorption by hydroxide. The corrected apparent quantum yields (Φ_app, PFAS, corr_) were significantly lower than those calculated via eqs 1 and 2: 0.0002 (PFBS), 0.0008 (PFOS), 0.0057 (PFOA), and 0.0068 (PFBA) (Table S16). At pH 9, there is insufficient overlap of KrCl* emission and the hydroxide absorption band (Figure S27) to enable e_aq_^–^ production via hydroxide photolysis, explaining the lack of PFBS and PFOS degradation at pH 9 under N_2_ purging.

Photolytic production of e_aq_^–^ by hydroxide also explains the increase in Φ_app,PFAS_ observed for PFCAs between pH 12 (our study) and pH 8.5 (Xin et al.^18^) in the presence of dissolved oxygen. At pH 12, direct photolysis via photodecarboxylation^18^ likely occurs concomitantly with e_aq_^–^-based destruction.

In the absence of dissolved oxygen at pH 12, oxidative radicals produced by hydroxide photolysis (^•^OH and O^•–^) may play a beneficial role. One pathway for e_aq_^–^ reaction with PFAS is H/F exchange, which yields polyfluorinated structures more resistant to e_aq_^–^ reduction.^22^ It is well-known that ^•^OH reaction with polyfluorinated compounds converts them to perfluorinated ones;^33,34^ thus, establishing subsequent reactivity with e_aq_^–^.

Additional support for the proposed mechanism comes from degradation experiments conducted using MCAA as an e_aq_^–^ probe. MCAA remains fully deprotonated (p*K*_a_ = 2.86)^35^ in the investigated pH range. Under 222 nm irradiation (*E*_0_ = 1.95 mJ s^–1^ cm^–2^), the pseudo-first-order rate constants (*k*_obs_) for photodegradation of 20 μM MCAA at pH 7 and 9 were 0.0224 and 0.021 min^–1^, which are identical within experimental error (Table S18). Chloride recoveries at pH 9 or lower were typically less than 50%. At pH 12, *k*_obs_ in the absence of sulfite (*k*_obs, OH^–^_) increased significantly to 0.157–0.263 min^–1^. This increase, coupled with the higher chloride recovery (67%), provides strong evidence for e_aq_^–^ generation via hydroxide photolysis at pH 12, whereas MCAA degradation at pH 9 and lower occurs by direct photolysis.

The apparent quantum yield of e_aq_^–^ from hydroxide at pH 12 (Φ_app, *e*_aq_^–^_) was calculated by assuming that MCAA acts as the primary e_aq_^–^ scavenger, neglecting reactions with H^+^ (due to high pH) and residual O_2_ (due to N_2_ purging). Although the calculated scavenging capacity of residual O_2_ (estimated [DO]_0_ ∼ 0.2 mg L^–1^)^36^ in N_2_-purged solution was significant (1.2 × 10^5^ s^–1^) compared to scavenging capacity of MCAA (2–8 × 10^4^ s^–1^), it was excluded for consistency with previous published models in N_2_-purged solution.^20,37^ The calculated Φ_app, e_aq_^–^_ for OH^–^ was 0.158 ± 0.014 (Table S18), which is 41% higher than the one literature value available of 0.112 measured at 193 nm excitation.^27,38^ For comparison, the literature value for Φ_e_aq_^–^_ from sulfite at 254 nm is 0.116 ± 0.002.

Future research is needed, ideally using laser flash photolysis, to verify the quantum yield of e_aq_^–^ at 222 nm excitation. Assuming Φ_*app*, *e*_*aq*_^–^_ values of 0.158 and 0.116 for hydroxide and sulfite, we calculated the steady state e_aq_^–^ concentrations ([e_aq_^–^]_ss_) at pH 12 with 45 μM MCAA for the UV only system to be 2.87 × 10^–12^ M, almost 1 M, almost an order of magnitude lower than for 5 mM sulfite, 1.08 × 10^–11^ M (additional details are provided in Text S12). Notably, in the presence of sulfite, [e_aq_^–^]_ss_ at pH 12 was 13.8-fold higher than that observed at pH 7 by Yin et al.^20^ The fluence-normalized steady-state concentration ([e_aq_^–^]_ss_/*E*_0,_ M cm^2^ s mJ^–1^) at pH 12 was 3.8-fold higher than at pH 7 (at *E*_0_ = 0.54 mJ cm^–2^ s^–1^).

We also investigated the impact of methanol, an ^•^OH scavenger, on defluorination of PFOA and PFOS at pH 12 in the absence of sulfite. As ^•^OH rapidly quenches e_aq_^–^ (*k* ≈ 3 × 10^10^ M^–1^ s^–1^),^28^ [e_aq_^–^] generated from hydroxide photolysis may be limited. Although the rate constant for quenching of ^•^OH by methanol (CH_3_OH + ^•^OH → ^•^CH_2_OH + H_2_O, *k* = 1.9 × 10^9^ M^–1^ s^–1^)^28^ is lower than that for ^•^OH quenching by e_aq_^–^, methanol can effectively scavenge ^•^OH at sufficiently high concentrations.^39^ The addition of 5 mM methanol to solutions of 25 μM PFOA or PFOS, 10 mM NaOH (pH 12), and saturated with N_2_ increased PFOS defluorination to 66 ± 1.8% compared to 35.8 ± 2.9% in the absence of methanol after 4 h of 222 nm irradiation (see Figure S20). In contrast, PFOA defluorination was unaffected by methanol addition (Figure S20). This observation is consistent with our hypothesis that PFCAs undergo both direct photolysis and e_aq_^–^-mediated degradation under UV only conditions at pH 12, whereas PFSAs are primarily degraded via reactions with e_aq_^–^ generated indirectly during photolysis of hydroxide.

### 3.4 UV_222_/Sulfite Kinetics at pH 12

Table 1 summarizes the fluence-normalized rate constants and defluorination percentages for the degradation of individual solutions of PFOA, PFBA, PFOS, and PFBS in the UV_222_/sulfite system at pH 12 with 10 mM sulfite. Kinetic profiles of parent compound loss and defluorination along with first-order rate constants, both in a time- and fluence-normalized basis, are shown in Figures S13 and S14. The presence of 10 mM sulfite increased the fluence-normalized rate constants for all four PFAS by 13- to 25- fold relative to the no sulfite condition at pH 12 under 222 nm irradiation. With 10 mM sulfite, the time required to achieve ∼100% defluorination was reduced from approximately 6 to 2 h for PFCAs and from 24 to 2 h for PFOS. In contrast, near-complete defluorination of PFBS was observed in 48 h, regardless of sulfite presence; however, sulfite addition increased the fluoride formation rate during the first 4 h from 0.003 to 0.016 mM h^–1^. These results indicate that enhanced generation of e_aq_^–^ via sulfite substantially accelerates defluorination at pH 12.

**Table 1:** Time- and Fluence-Based Rate Constants and Defluorination Percentages for PFCAs and PFSAs in UV/Sulfite-ARP

parent compound	*k* (h^–1^)^a^	*k*_*F*_ (cm^2^ J^–1^)^b^	deF (%)^c^	deF_*F*_ (% cm^2^ J^–1^)^d^	*k*_*F*_ literature at 222 nm (cm^2^ J^–1^)
PFOA	13.67	2.52	104 (2 h)	9.49 (2 h)	0.106^e^
PFBA	14.16	2.61	99.5 (2 h)	9.12 (2 h)	
PFOS	2.77	0.51	101 (24 h)	0.78 (24 h)	0.13^f^
PFBS	0.39	0.07	83.8 (24 h)	0.64 (24 h)	0.01^f^

a *k* is the time-based first-order rate constant (h^–1^). [PFAS]_0_ = 25 μM, [Na_2_SO_3_] = 10 mM, and pH 12.

b *k*_*F*_ is the fluence-normalized *k* (cm^2^ J^–1^) where the normalization is with respect to incident fluence rate.

c defluorination percentage (deF %) calculated at a given reaction time.

d The fluence-normalized deF% (deF_*F*_) was calculated by dividing deF% to the corresponding incident fluence.

e From ref.;^20^ Conditions: [PFOA]_0_ = 2 μM, sulfite 1 mM, and pH 7.

f From ref.;^19^ Conditions: [PFAS]_0_ = 8 μM, sulfite 1 mM, and pH 10. Detailed experimental conditions for the published data are reported in references.^19,20^

As summarized in Table 1, rate constants normalized to incident fluence rate at pH 12 are larger than those for the lower pH values reported in the literature (pH 7 or pH 10),^19,20^ ranging from a 4-fold (PFOS) to 24-fold (PFOA) increase. These prior studies employing pH 7 and 10 utilized a lower concentration of sulfite (1 mM) than in our study (10 mM). During UV/sulfite treatment of PFOS at 222 nm, Xin et al. showed that the degradation rate constant was largest at 1 mM sulfite, with subsequent decreases at higher concentrations.^19^ If a 1 mM sulfite dosage is also optimal at pH 12, then the enhancement in fluence-normalized degradation at pH 7–10 relative to pH 12 are likely larger than the 4- to 24-fold described above.

### 3.5 Comparison of 222 and 254 nm for PFAS Destruction in Complex Systems

Two recent studies have demonstrated larger fluence-normalized rate constants of various PFAS under 222 nm irradiation relative to 254 nm in the UV/sulfite-ARP.^19,20^ However, these experiments were conducted without real water matrix components (e.g., DOM, nitrate) or with each parameter tested individually. To address this knowledge gap, experiments were performed in two different matrices: (i) ROC spiked with mg/L of short and long chain perfluoroalkyl acids and (ii) a highly diluted AFFF. The impact of competition for e_aq_^–^ among mixtures of PFAS was evaluated by comparing parent compound rate constants and defluorination for single-solute solutions and mixtures under UV_222_ only and UV_222_/sulfite treatment.

#### 3.5.1 PFAS Mixture Spiked into Reverse Osmosis Concentrates

We studied the defluorination kinetics of a four PFAS mixture (PFOA, PFBA, PFOS, and PFBS spiked at 25 μM) in ROC containing high levels of DOM (52.1 mg_C_ L^–1^), nitrate (2.6 ± 0.029 mM), and low UV transmittance (<1% at 222 and ∼12% at 254 nm) (see Table S3). Native PFAS in ROC were measured in our previous work and found to be ≲1 μg L^–1^; while PFOA, PFBA, PFOS and PFBS were below detection limits.^4^ The influence of reactor geometry on photochemical kinetics is important when comparing results across different wavelengths, although it was not addressed in this study. To facilitate comparison, we employed the same conditions in this work as Fennell et al.^4^ (pH 12, 50 mM sulfite). Detailed experimental conditions are shown in Table S4 and the replicability of PFAS defluorination experiments is presented in Figure S18. Solution pH and inorganic anions were monitored during degradation experiments. Solution pH remained stable during ROC irradiation, with average ± standard deviations of 12.1 ± 0.13 and 12.0 ± 0.2 across all time points of UV-only and UV/sulfite ARP treatment, respectively (Figure S26).

Figure 4 demonstrates a marked increase in the deF% achieved at lower fluence in the UV_222_/sulfite-ARP relative to 254 nm. For example, irradiation for 24 h resulted in a 2.3-fold larger deF% at a 3-fold lower fluence. Near-quantitative defluorination of the four-PFAS mixture was observed by 48 h (276 J cm^–2^) at 222 nm, whereas treatment with 254 nm for 48 h (814 J cm^–2^) resulted in 71% defluorination.^4^ When normalizing the deF% at 24 h to incident fluence, the photon-based efficiency of defluorination at 222 nm was 6.3 times greater than at 254 nm (0.44% cm^2^ J^–1^ vs 0.071% cm^2^ J^–1^). At 48 h, fluence-normalized deF% was 3.9 times greater at 222 nm than 254 nm (0.344% cm^2^ J^–1^ vs 0.087% cm^2^ J^–1^). It is also notable that high deF% (∼80%) is achieved in the UV_222_ treatment at pH 12 without sulfite addition.

**Figure 4:**
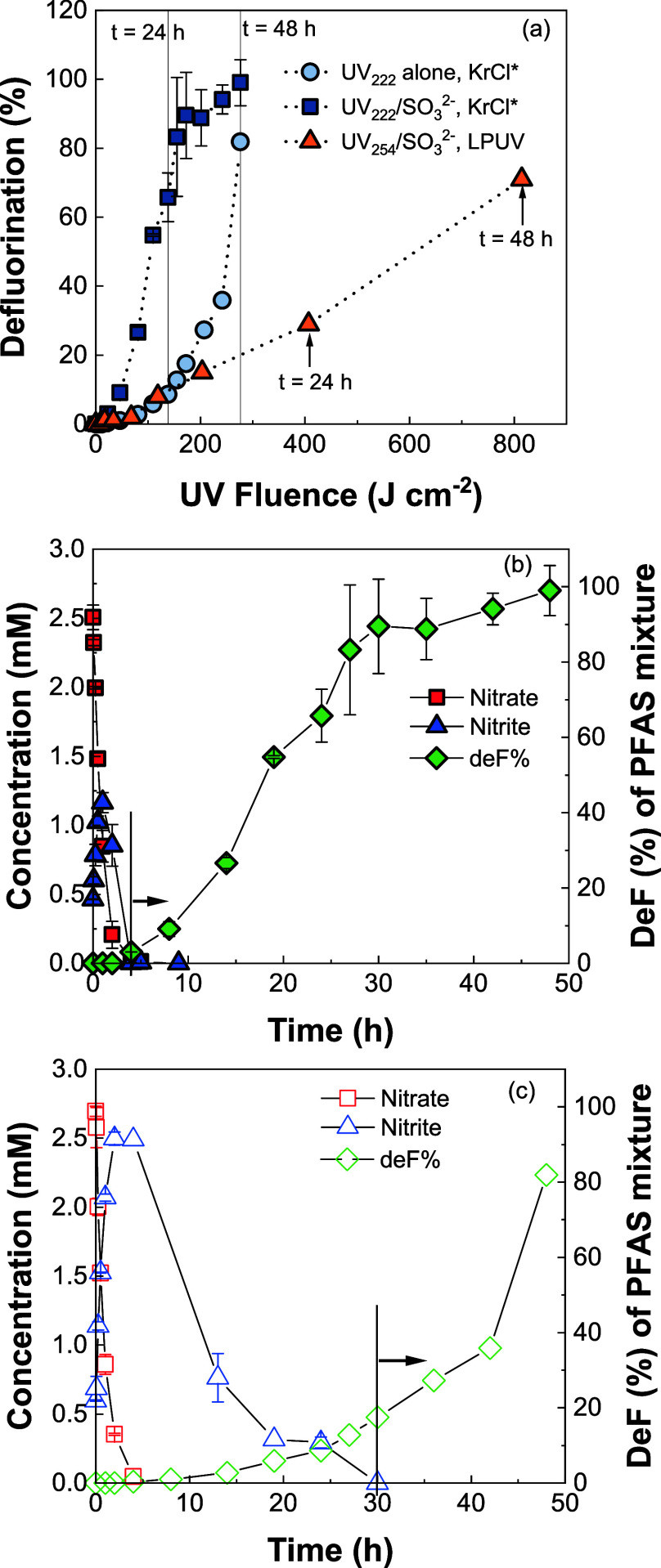
Defluorination of PFAS mixture in ROC treated by UV_222_/sulfite ARP and UV_254_/sulfite ARP (a) and concentrations of nitrate and nitrite along with PFAS defluorination in ROC by UV_222_/sulfite ARP (b) and UV_222_ only (c). Conditions: [PFOA]_0_ = [PFBA]_0_ = [PFOS]_0_ = [PFBS]_0_ = 25 μM, [Na_2_SO_3_]_0_ = 50 mM, N_2_-purged, and pH 12. Concentrations of nitrate and nitrite at 222 nm (b and c) were determined in experiments 6–7 and 9–10, and the deF% was measured in experiments 1–3 (Table S4). Data for UV_254_/sulfite ARP and additional experimental conditions are from Fennell et al.^4^ Solution volumes were ∼17 mL for 222 nm and ∼570 mL for 254 nm. The incident UV fluence rates range from 3.00 to 3.63 × 10^–9^ mol photons cm^–2^ s^–1^ at 222 nm and from 0.99 to 1.21 × 10^–8^ mol photons cm^–2^ s^–1^ at 254 nm.^4^

Although prior comparisons of KrCl* and LP-Hg lamps have employed incident fluence rate, calculations using average UV fluence rate, which incorporate the water factor (WF), show an even greater enhancement at 222 nm. The incident fluence rate measured with iodide-iodate actinometry in both 222 and 254 nm systems was employed to calculate the average UV fluence rate as *E*_0_ × WF where $\mathrm{WF}=\frac{1-10^{-al}}{2.303al}$ , *a* is the absorbance (cm^–1^), and l is the effective path length (cm). Note that the absorbances of 0.533 and 0.014 cm^–1^ at 222 and 254 nm, respectively, were measured on a 121-fold diluted ROC sample spiked with 50 mM sulfite, and the dilution factor was used to calculate the actual absorbance in the reactor. The corresponding WF for 222 and 254 nm are 0.0066 and 0.11, respectively, resulting in average fluence rates of 0.0129 and 0.501 mJ cm^–2^ s^–1^. After 48 h of irradiation in the UV/sulfite system, the defluorination reached 99% at 222 nm and 71% at 254 nm. When normalized to the inputted UV fluence at 48 h (average fluence rate × 48 h), the fluence-normalized defluorination was 44.3% cm^2^ mJ^–1^ and 1.14% cm^2^ mJ^–1^, for 222 and 254 nm, respectively. Based on this normalization, 222 nm irradiation showed an approximately 40-fold enhancement in performance relative to 254 nm.

Because the absorbance at both 222 and 254 nm will change over the course of the 48 h irradiation, the results shown here offer only a first approximation on the impact of WF. In contrast, normalizing to incident fluence rate does not require knowledge of how solution absorbance changes with time.

The improved defluorination at lower fluence in the 222 nm UV/sulfite system, compared to the 254 nm UV/sulfite system (Figure 4a) is attributable to the additional pathways enabled by 222 nm photons, such as direct photolysis of PFCAs and activation of additional e_aq_^–^ sources (i.e., hydroxide), the increased molar absorptivity of sulfite at 222 nm (Table S17), and the potential for improved kinetic removal of inhibiting water matrix components (e.g., nitrate). Previous studies have demonstrated that the larger ε of sulfite at 222 nm results in a greater concentration of e_aq_^–^.^20^ In ROC, sulfite absorbs a larger fraction of the absorbed photons at 222 nm (∼83%) relative to 254 nm (∼47%, Table S22); increasing the fraction of photons capable of producing e_aq_^–^.

Nitrate can lower e_aq_^–^ concentrations by shielding incoming photons and scavenging e_aq_^–^.^28^ Indeed, comparison of the time profiles for nitrate, nitrite, and deF% demonstrate that PFAS destruction is highly limited until these species are abated (Figure 4b). In the UV/sulfite system at pH 12, both nitrate and nitrite are completely degraded in ∼2 h (∼12 J cm^–2^) under 222 nm irradiation, whereas both species were not abated until ∼8 h (∼130 J cm^–2^) at 254 nm.^4^ First-order rate constants for nitrate loss in ROC were 1.05 and 0.60 h^–1^ at 222 and 254 nm, respectively (Figure S25). To evaluate the extent of direct photolysis, quantum yields for nitrate loss were measured in ultrapure water and used to calculate the expected first order photodegradation rate in ROC based on the initial nitrate concentration and measured absorbance (Text S14). The calculated value of 0.217 h^–1^ is lower than the observed rate constant for nitrate decay in ROC of 1.05 h^–1^. This suggests that ∼21% of the observed nitrate decay is due to direct photolysis, with the remaining 79% attributable to degradation by e_aq_^–^.

The inhibitory effect of nitrate was also observed during UV_222_ treatment of ROC in the absence of sulfite (Figure 4c), with defluorination being limited to <20% up until ∼30 h and increasing to ∼80% by 48 h. The rapid increase in defluorination was concomitant with nitrite abatement (Figure 4c). The markedly slower degradation of nitrite in the absence of sulfite is consistent with e_aq_^–^-mediated degradation being the dominant pathway for removal of nitrite in ROC rather than direct photolysis.

#### 3.5.2 Aqueous Film Forming Foam

Another relevant matrix for testing UV_222_ technology is AFFF, which contains a complex mixture of PFAS. Here, we employed UV_222_/sulfite for the degradation of PFAS in an AFFF previously characterized by ^19^F-NMR, having a total organic fluorine content of 14.5 g L^–1^ and whose quantifiable PFAS was dominated by PFSAs.^23^ This same AFFF was previously studied in the UV/sulfite system at 254 nm by Tenorio et al.^23^ The UV fluence rate in this study was measured as 3.1 × 10^–9^ mol photons cm^–2^ s^–1^ at 222 nm, compared to 1.0 × 10^–8^ mol photons cm^–2^ s^–1^ at 254 nm reported by Tenorio et al.^23^ By matching the 1:60,000 AFFF dilution and experimental conditions employed by Tenorio et al. (10 mM sulfite, pH 9.5), we can directly compare the time-based defluorination (Figure 5). The maximum defluorination observed under 222 nm irradiation was ∼ 86% at 48 h, while Tenorio et al. measured a 53% defluorination at 49 h under 254 nm.^23^ The UV fluence-normalized deF% was 0.3% cm^2^ J^–1^ at 48 h under 222 nm, which is 4.6-fold higher than the value at 254 nm (see Table S25).

**Figure 5:**
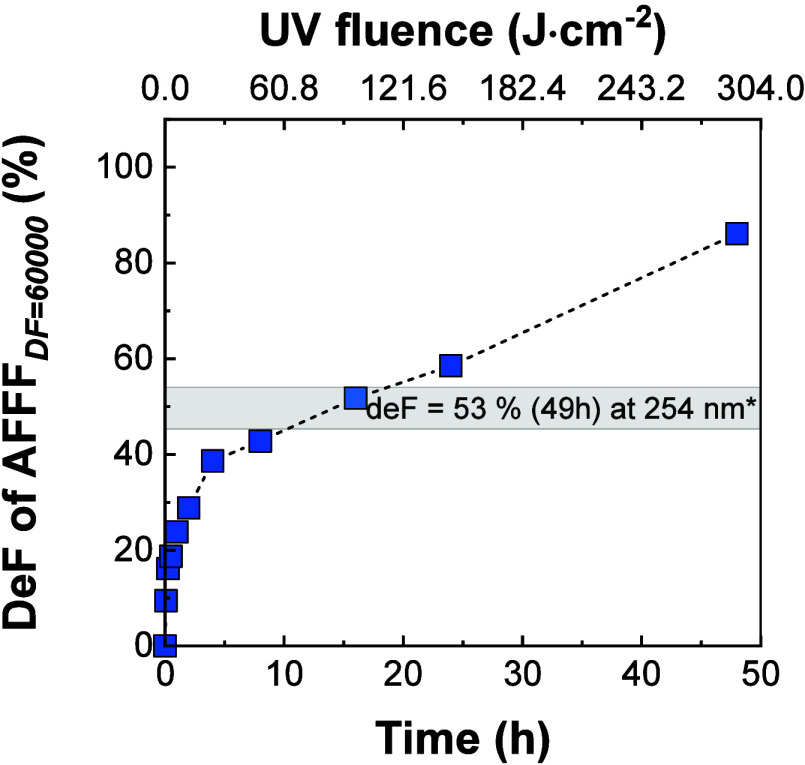
Percentage of total fluorine released as fluoride from diluted AFFF during the UV/sulfite ARP. Conditions: AFFF (1-to-60,000 dilution), [Na_2_SO_3_]_0_ = 10 mM, pH_0_ = 9, and [NaHCO_3_]_0_ = 5 mM, and incident UV fluence rate at 222 nm = 3.1 × 10^–9^ mol photons cm^–2^ s^–1^ (1.69 mJ cm^–2^ s^–1^). Solution volumes were ∼17 mL for 222 nm and ∼575 mL for 254 nm.^23^ Defluorination was calculated as the ratio of released F^–^ to total organic fluorine. Defluorination of 53% in 49 h at 254 nm* (1.0 × 10^–8^ mol photons cm^–2^ s^–1^) is from Tenorio et al.^23^

There are limitations in the above comparison, as it is based on different studies that utilized different photochemical reactors and treatment volumes. However, the results are consistent with results from the ROC (Section 3.5.1) and previous studies suggesting that 222 nm-based UV-ARP are more efficient than at 254 nm irradiation.^19,20^ Other studies employing UV/sulfite at 254 nm have observed lower amounts of defluorination (<40%) during treatment of AFFF under more extreme conditions such as elevated temperature or ∼100 mM sulfite.^40,41^ Preliminary tests were also conducted at a lower dilution factor (1:2000), demonstrating >70% defluorination of AFFF in the presence of 20 mM sulfite at both pH 9.5 and 12.^42^ More in-depth understanding of PFAS kinetic decay, factors influencing treatment of AFFF, and defluorination efficiency in different AFFF sources, is necessary and will be the subject of a forthcoming study.

#### 3.5.3 UV_222_ Only Treatment of PFAS Mixtures

Prior studies on UV_222_-based PFAS degradation have reported the degradation kinetics of individual PFAS in deionized water.^18^ Although our results for PFAS mixtures spiked into ROC indicate that high levels of defluorination can be achieved based on the known initial concentration of spiked compounds, we desired to investigate whether there was any inhibition in the decay kinetics of PFAS mixtures relative to individual compounds. To test the potential for inhibition, UV_222_ only and UV_222_/sulfite experiments were conducted in deionized water for a four-PFAS mixture containing PFBS, PFOS, PFBA, and PFOA. Single solute experiments were also performed. Time courses and first-order rate constants (Table S14) indicate that parent compound degradation is impeded in the mixture compared to single solute experiments for both UV only and UV/sulfite conditions (Figure S15). Importantly, in the UV_222_/sulfite system, all PFAS were degraded to near detection limits, including PFBS, and ∼100% defluorination was reached (Figure S16b). In contrast, UV_222_ only treatment of the PFAS mixture exhibited delayed degradation of PFOS and minimal degradation of PFBS (Figure S16a). In the absence of sulfite, first-order rate constants decreased by approximately 20% for PFOA, 59% for PFBA, 58% for PFOS, and 93% for PFBS when comparing single-compound systems to the mixture (see Table S14). In the presence of sulfite, a similar effect was observed. The rate constants decreased by approximately 56% for PFOA, 76% for PFBA, 88% for PFOS and 83% for PFBS in the mixture system. While all compounds were impacted, shorter-chain PFAS were the most inhibited.

These results clearly demonstrate that competition among PFAS for reactive species occurs in mixed systems and leads to reduced degradation rates. Although we are unable to fully elucidate the mechanism of this inhibition within the scope of this paper, light shielding by individual PFAS is an unlikely explanation given that at pH 12, hydroxide will be the major light absorber. Future work on this topic is necessary to fully develop KrCl* lamp’s potential for UV-ARP treatment of PFAS.

## 4 Implications for UV-Advanced Reduction Processes and PFAS Treatment

We have demonstrated that 222 nm-emitting KrCl* lamps can degrade short- and long chain perfluoroalkyl acids in the presence and absence of sulfite at pH 12. This work expands the parameter space of recent studies on UV_222_ treatment of PFAS^18–20^ by focusing on pH 12, which has been identified as the most efficient pH for PFAS destruction in UV-ARP at 254 nm.^22^ Although true direct photolysis occurs for PFCAs at pH 12, degradation of PFSAs in the absence of sulfite is most likely due to photoproduction of e_aq_^–^ from hydroxide. This unanticipated pathway is analogous to the recently identified role of dissolved oxygen-induced reactive oxygen species hypothesized to be responsible for the enhanced “direct photolysis” of organic micropollutants in UV_222_ systems.^21^ Unlike previous studies focused on single compound systems, this work also tested the kinetics and defluorination of PFAS mixtures in complex matrices. An important conclusion from this work is that the UV_222_/sulfite system shows improved fluence-normalized fluoride release during treatment of complex water matrices representative of real-world scenarios. It is demonstrated that this enhancement can be partly explained by faster degradation of nitrate, a ubiquitous e_aq_^–^ scavenger, under 222 nm irradiation.

Although the results presented here and elsewhere appear promising, it is not immediately apparent that KrCl* lamps will supersede LP-Hg lamps in UV-ARP. In addition to the potential benefit of faster defluorination kinetics shown above, use of 222 nm may allow UV-ARP to operate at lower doses of sulfite and lower pH. For example, if the contamination is largely due to PFCAs, neutral pH and oxygenated conditions could be used without sulfite, which could simplify process design for certain waste streams. Preliminary experiments with AFFF suggest that defluorination in the UV_222_/sulfite system is not pH dependent and that appreciable defluorination (up to ∼ 30%) occurs under mild conditions (pH 9.5 in the presence of dissolved oxygen).^42^ A downside of KrCl* lamps is their lower wall plug efficiency (5–15%) compared to low-pressure mercury lamps (30–38%),^20,43^ which may offset the benefit of more rapid parent compound degradation and defluorination at 222 nm. However, a prior study reported 10-fold larger electrical energy per order for degradation of MCAA in the UV_222_/sulfite system in deionized water, even when differences in wall plug efficiency were considered.^20^ An additional concern worth noting is the almost certain formation of nitrated byproducts (e.g., nitrophenols) produced during irradiation of nitrate-containing wastes. Although quantifying the formation of nitrophenols was not within the scope of our study, we posit that their lifetime is less than the typical irradiation time for these experiments (24–48 h) due to nitrophenol’s high reactivity with e_aq_^–^ (3.0 × 10^10^ M^–1^ s^–1^ for nitrobenzene; 4.4 × 10^10^ M^–1^ s^–1^ for 4-nitrophenol).^44,45^ Future studies investigating optimization for various PFAS-impacted matrices, formation and decay of byproducts, and comparison to 254 nm systems in identical reactor geometries are needed to fully elucidate the potential benefits of KrCl* lamps in UV-ARP.

## Supplementary Material



## Data Availability

Data will be made available upon reasonable request to the authors.

## Notes

Additional experimental details, materials and methods, and data tables and figures (PDF)
