# Supplementary material for: Degradation of Perfluoroalkyl Acids under 222 nm Irradiation in the Presence and Absence of Sulfite
Source: ACS ES T Eng. 2026 May 2;6(6):1849–58. doi: 10.1021/acsestengg.6c00161 (PMC13270529; doi:10.1021/acsestengg.6c00161)
Supplement: Supplementary file 1 [file ee6c00161_si_001.pdf]

Supporting Information for

Degradation of Perfluoroalkyl Acids under 222 nm Irradiation in the Presence and Absence of Sulfite

Bahngmi Jung*, and Garrett McKay*

Zachry Department of Civil & Environmental Engineering, Texas A&M University,

College Station, TX, USA 77845

Number of Pages: 43

Number of Sections:14

Number of Tables:25

Number of Figures:28

Corresponding authors: Bahngmi Jung, angela-b.jung@tamu.edu;

Garrett McKay, gmckay@tamu.edu, 979.458.6540

Table of Contents

Text Sections

Text S1. Chemicals 5

Text S2. Reverse osmosis concentration (ROC) pretreatment 7

Text S3. Determination of the UV fluence rate 9

Text S4. Fluoride measurements 10

Text S5. Inorganic anion measurement 11

Text S6. Sulfite measurement 11

Text S7. PFAS measurements 12

Text S8. Individual PFAS degradation at pH 12. 15

Text S9. Behavior of PFAS degradation in a mixture solution 23

Text S10. Quantum yield calculations for PFAS UV photolysis 24

Text S11. Reproducibility of $PFAS_{mixture}$ defluorination experiments 28

Text S12. Mechanistic evidence for hydrated electron generation from OH^- photolysis 30

Text S13. Estimation of light absorption fraction of major constituents in ROC 34

Text S14. Quantum yield for nitrate decay and nitrite formation from nitrate at 222 nm 36

Tables

Table S1. List of chemicals used in this study 5

Table S2. PFAS chemicals used in experiments 6

Table S3. Water quality parameters for OCWD ROC with cation exchange pretreatment. 7

Table S4. Experimental conditions of PFAS defluorination using UV-ARP. 8

Table S5. Recovery of four native PFAS mixture standards at 22.5 ng/mL 12

Table S6. Recovery of 0.1 μM $PFAS_{mixture}$ diluted solution in experiments 1-3. 14

Table S7. Concentrations of the $PFAS_{mixture}$ and total F^- released in a reactor. 14

Table S8. Recovery of 0.1 μM $PFAS_{mixture}$ diluted solution in experiments 4-8. 14

Table S9. Recovery of 0.1 μM individual PFAS diluted solution in experiments 11-14 14

Table S10. Rate constant and defluorination efficiency of PFCAs. 16

Table S11. Electrical energies per order for PFAS degradation by UV-only and UV/sulfite ARPs. 16

Table S12. Calculated absorption coefficient (a) and water factor (WF) for PFAS solution. 18

Table S13. Experimental conditions for light control 18

Table S14. Comparison of time-based rate constants of single and mixed PFAS. 23

Table S15. Literature pK_a values, and measured molar absorptivity, rate constants, and quantum yields. .25

Table S16. Apparent quantum yield corrected by OH^- light absorption 27

Table S17. Molar absorptivity and quantum yield of water matrix components at 222 and 254 nm. 28
Table S18. Experimental conditions of MCAA degradation in the absence of sulfite under 222 nm. 31
Table S19. Comparison of $[e_{aq}^{-}]_{ss}/E_0$ for MCAA degradation with literature values. 32
Table S20. Calculation of EE/O for MCAA degradation and comparison with literature data. 32
Table S21. Light absorption calculations on 121-fold diluted ROC...34
Table S22. Calculated light absorption fraction of major constituents in ROC 34
Table S23. Rate constants and quantum yields for nitrate decay by UV_{222} photolysis. 36
Table S24. Initial rates and quantum yield of nitrite formation from nitrate at 222 nm. 37
Table S25. Fluence-normalized defluorination of AFFF at 222 nm versus 254 nm. 40

Figures

Figure S1. The illustration of the experimental setup. 10
Figure S2. Calibration curve for ISE (a) and comparison of $[F^-]$ between IC and ISE (b). 10
Figure S3. Sulfite concentrations during $PFAS_{mixture}$ decay in ROC by UV_{222}/sulfite-ARP. 11
Figure S4. Photodegradation and defluorination percentages of PFOA (a), PFBA (b), PFOS (c), and PFBS (d) in deionized water at 222 nm. 15
Figure S5. Electrical energy per order (EE/O) for PFAS degradation by UV_{222}-only and UV_{222}/sulfite ARP.. 17
Figure S6. The percentages of degradation and defluorination of PFCAs under 222 nm irradiation.........18
Figure S7. Degradation of monochloroacetic acid under 222 nm. 19
Figure S8. Degradation products of PFOA by UV_{222} photolysis...19
Figure S9. PFBA degradation products by UV_{222} photolysis. 20
Figure S10. Degradation and defluorination of PFOS and PFBS. 20
Figure S11. Fluence-normalized defluorination for four individual PFAS compounds at 222 nm dependent on the solution pH and N_2 saturation. 21
Figure S12. Solution pH changes during degradation of individual PFAS compounds under UV-only conditions. 21
Figure S13. Degradation and defluorination of PFAS parent compounds by UV_{222}/sulfite ARP. 22
Figure S14. Kinetics of PFAS degradation in the UV_{222}/sulfite ARP.. 22
Figure S15. Time-based rate constants of single vs. mixed PFAS by UV only (a) and UV_{222}/sulfite ARP (b). 23
Figure S16. Degradation and defluorination of $PFAS_{mixture}$ under UV_{222}-only (a) and UV_{222}/sulfite ARP (b) in deionized water.. 24
Figure S17. $[F^-]$ versus time during UV_{222} photolysis of individual PFAS compounds. 26

Figure S18. Defluorination of the $PFAS_{mixture}$ in ROC by UV_{222}–only and UV_{222}/sulfite ARP. 28
Figure S19. Molar absorptivity of sulfite, iodide, nitrate, and nitrite, and PFAS at 222 and 254 nm.........29
Figure S20. Effect of addition of 5 mM methanol on PFAS defluorination. 33
Figure S21. Total absorbance and estimated absorbance from sulfite concentrations for 121-fold diluted ROC at 222 nm...35
Figure S22. UV-visible spectra in deionized water and ROC samples. 35
Figure S23. Nitrate (a) and nitrite (b) profiles during $PFAS_{mixture}$ decay in ROC. 36
Figure S24. Nitrate decay and nitrite formation by UV222 photolysis. 37
Figure S25. Nitrate and nitrite profiles at different wavelengths (222 and 254 nm) during $PFAS_{mixture}$ treatment in ROC..38
Figure S26. Time profiles of sulfite (a), chlorate (b), chloride (c), bromide (d), formate (e), and pH (f) during $PFAS_{mixture}$ decay in ROC by UV_{222} photolysis and UV_{222}/S-ARP...39
Figure S27. Absorption spectra of NaOH at different concentrations in aqueous solutions. 40
Figure S28. Absorbance of NaOH solutions at 222 nm as a function of pH. 40

Text S1. Chemicals

Most chemicals used in this study were purchased from Sigma-Aldrich or VWR. Per- and polyfluoroalkyl substances (PFAS) standards and surrogates were obtained from Wellington Laboratories LLC (Table S1 and S2). Solutions, except for those containing PFAS, were prepared by dissolving the chemical salts in ultrapure water (18.2 MW-cm, Barnsted Water Purification System) and stored at 4°C. Sodium sulfite solutions were freshly prepared for each UV-ARP experiment by dissolving sodium sulfite salt in deoxygenated deionized water. In the UV/sulfite-ARP, sulfite rapidly deoxygenates water through a thermal reaction,[1] obviating the need for nitrogen purging during application of UV/sulfite-ARP in real world scenarios. For this study, we desired to create similar conditions between UV photolysis and UV/sulfite-ARP experiments, which is why all samples were purged with N_2. All experimental solutions were deoxygenated by purging with nitrogen gas (industrial grade, Airgas Company) for 30-120 minutes. Following this, the sulfite solution was further purged with nitrogen gas for an additional 5-minute then transferred to an anaerobic chamber (97% N_2 and 3% H_2, Coy Laboratory Products, Inc.).

Table S1. List of chemicals used in this study

Chemical	CAS No. or Lot No.	Purity
1000 ppm 7 anion calibration standard solution	IV-STOCK-59-125mL	ISO17034
5,5’dithiobis (2-nitrobenzoic acid)	69-78-3	99%
Ammonium hydroxide (28-30% NH_3)	1336-21-6	ACS reagent
Boric acid	10043-35-3	99.97%
Buffer, precision reference standard pH10	1602-16	ISO17025
Buffer, precision reference standard pH4	1502-16	ISO17025
Buffer, precision reference standard pH7	1552-16	ISO17025
Hydrochloric acid (2 N)	7647-01-0	ACS reagent
Inorganic cation calibration standard solution	IC-SCS1-125mL	ISO17034
Methanesulfonic acid	75-75-2	^{3}99%
Methanol	67-56-1	LC/MS grade
Potassium chlorate	25572-100g	^{3}99%
Potassium chloride	744-40-7	>99%
Potassium iodide	7681-11-0	^{3}99%
Potassium phosphate dibasic	11/4/7758	98%
Potassium phosphate monobasic anhydrous	7778-77-0	99%
Sodium bicarbonate	144-55-8	>99.7%
Sodium bromate	7789-38-0	^{3}99%
sodium chloroacetate	3926-62-3	98%
Sodium fluoride	7681-49-4	>99%
Sodium iodate	7681-55-2	^{3}99%
Sodium nitrate	7631-99-4	^{3}99%
Sodium nitrite	7632-00-0	^{3}97%
Sodium sulfite	7757-83-7	^{3}98%
Sulfuric acid (95-98%)	7664-93-9	ACS reagent

Table S2. PFAS chemicals used in experiments

Compounds	CAS number or Product code	Purity	Provider
	Analytes of [a]PFAS$_{mixture}$		
Heptadecafluorooctanesulfonic acid potassium salt (PFOS)	2795-39-3	^{3}98%	Sigma-Aldrich
Sodium perfluorooctanoate (PFOA)	335-95-5	97%	Alfa Aesar
Heptafluorobutyric acid (PFBA)	375-22-4	^{3}97.5%	Sigma-Aldrich
Nonafluorobutane-1-sulfonic acid (PFBS)	375-73-5	ISO9001	Sigma-Aldrich
	Native PFAS standards		
Perfluoro-n-octanoic acid (PFOA)	335-67-1(PFOA0523)	>98%	Wellington Lab.
Perfluloro-n-butanoic acid (PFBA)	375-22-4(PFBA0323)	>98%	Wellington Lab.
L-potassium perfluoro-1-butanesulfonate (L-PFBS)	29420-49-3(LPFBS0423)	>98%	Wellington Lab.
Sodium perfluoro-1-octanesulfonate (L-PFOS)	4021-47-0(LPFOS0523)	>98%	Wellington Lab.
	Surrogates		
Perfluoro-1-[13C8]octanoic acid	M8PFOA	>98%	Wellington Lab.
(Na)-Perfluoro[13C8]octanesulofonate	M8PFOS	>98%	Wellington Lab.
Perfluoro-n[2,3,4-13C3]butanoic acid	M3PFBA	>98%	Wellington Lab.
(Na)Perfluoro-1-[2,3,4-13C3]butanesulfonate	M3PFBS	>98%	Wellington Lab.

[a]PFAS$_{mixture}$ refers to a mixture of four PFAS compounds: PFOA, PFOS, PFBA and PFBS.

Text S2. Reverse osmosis concentration (ROC) pretreatment

All procedures used for ROC pretreatment were adapted from our previous work.[2] Reverse osmosis concentrate (ROC) for irradiation experiments were collected from the Orange County Water District (OCWD) Groundwater Replenishment System, which purifies highly treated wastewater for indirect potable reuse. Upon receipt, ROC was filtered through muffled (500 °C for 4 h) Whatman glass microfiber filters (GF/A, 1.6 mm), pre-rinsed with ultrapure water. UV-ARP experiments were conducted at high pH (~ pH 12) to enhance PFAS defluorination, as higher pH accelerate this process.[2] Previous studies indicated that high pH in OCWD ROC results in white precipitate formation, requiring removal of water hardness. In this study, water hardness (Ca^{2+} and Mg^{2+}) was removed using an ion exchange resin. Approximately 2 inches of iFilters water softener cation resin (MED-CAT-025, 8000 grain capacity) was packed into an Ace Glass Filter column (porosity B, 70-100 mm) with a 20 mm diameter and stopcock. The resin was rinsed with 150 mL of ultrapure water, followed by slow addition and 400 mL of OCWD ROC drainage. The first 25 mL of effluent OCWD ROC was discarded to remove residual ultrapure water. Before irradiation, the ROC sample pH was adjusted using 5 N NaOH and purged with nitrogen gas, then transferred to an anaerobic chamber.

Table S3. Water quality parameters for OCWD ROC with cation exchange pretreatment.

Parameters	OCWD ROC with filtering and cation exchange
DOC (mg C L^{-1})	52.1 [2]
NO_3^- (mM)	2.6 ± 0.029
NO_2^- (mM)	0.47 ± 0.007
Bromate (mgL^{-1})	BDL
Chlorate (mgL^{-1})	1566 ± 34
Fluoride (mgL^{-1})	4.8 ± 0.2
Bromide (mgL^{-1})	7.5 ± 0.1
Chloride (mgL^{-1})	2801 ± 17.2
Sodium (mgL^{-1})	3800
Potassium (mgL^{-1})	35
Magnesium (mgL^{-1} as $CaCO_3$)	6
Calcium (mgL^{-1} as $CaCO_3$)	2.0
pH	8.3 ± 0.1
A_{222nm} (cm^{-1}) of ROC at pH 12	>2
A_{254nm} (cm^{-1}) of ROC at pH 12	0.935

Table S4. Experimental conditions of PFAS defluorination using UV-ARP.

Exp. No.	Reaction conditions					Incident fluence rate (E_0) mol photons $\times cm^{-2} \times s^{-1}$	pH_0	Reaction time (h)
	$[PFAS]_0$ (mM)	$[Na_2SO_3]_0$ (mM)	wavelength (nm)	Water media	N_2 purging			
1	$PFAS_{mixture}$	50	222	ROC[b]	Y	3×10^{-9}	12.1	~48
2	$PFAS_{mixture}$	50	222	ROC	Y	3×10^{-9}	12.1	~48
3	$PFAS_{mixture}$	0	222	ROC	Y	3×10^{-9}	11.9	~48
4	$PFAS_{mixture}$	0	222	DIW	Y	3×10^{-9}	11.9	~48
5	$PFAS_{mixture}$	50	222	DIW	Y	3×10^{-9}	12.2	~48
6	$PFAS_{mixture}$	50	222	ROC	Y	3.63×10^{-9}	11.7	~48
7	$PFAS_{mixture}$	50	222	ROC	Y	3.63×10^{-9}	11.9	~48
8[a]	$PFAS_{mixture}$	50	222	ROC	Y	3.63×10^{-9}	11.9	~48
9	$PFAS_{mixture}$	0	222	ROC	Y	3.63×10^{-9}	12.2	~48
10	$PFAS_{mixture}$	0	222	ROC	Y	3.63×10^{-9}	12.1	~48
11	PFBA	0	222	DIW	Y	2.81×10^{-9}	12	~48
12	PFOA	0	222	DIW	Y	2.81×10^{-9}	12	~48
13	PFBS	0	222	DIW	Y	2.81×10^{-9}	12	~48
14	PFOS	0	222	DIW	Y	2.81×10^{-9}	12	~48
15	PFBA	0	222	DIW	N	2.1×10^{-9}	12	~48
16	PFBA	0	222	DIW	Y	2.92×10^{-9}	9	~48
17	PFOA	0	222	DIW	N	2.1×10^{-9}	12	~48
18	PFOA	0	222	DIW	Y	2.1×10^{-9}	9	~48
19	PFBS	0	222	DIW	N	2.81×10^{-9}	12	~48
20	PFBS	0	222	DIW	Y	2.81×10^{-9}	10	~48
21	PFBS	0	222	DIW	Y	3×10^{-9}	8	~48
22	PFOS	0	222	DIW	N	2.1×10^{-9}	12	~48
23	PFOS	0	222	DIW	Y	2.92×10^{-9}	10	~48
24	PFBA	10	222	DIW	Y	2.8×10^{-9}	12	~48
25	PFOA	10	222	DIW	Y	2.8×10^{-9}	12	~48
26	PFBS	10	222	DIW	Y	2.8×10^{-9}	12	~48
27	PFOS	10	222	DIW	Y	2.8×10^{-9}	12	~48

A 1 mM $PFAS_{mixture}$, as well as 1 mM individual PFOA, PFBA, PFOS, and PFBS stock solutions were spiked into the reactor to achieve an initial concentration of 0.025 mM. The $PFAS_{mixture}$ refers to a mixture of four PFAS compounds: PFOA, PFOS, PFBA and PFBS. [a]1 mM monochloroacetic acid (MCAA) was added in exp. 8. [b]ROC refers to a reverse osmosis concentrate water sample without pH buffer. [c]DIW refers to deionized water. Irradiation experiments included more than ten reaction times over a 48-hour period. The solution pH was adjusted to 12 or 8-10 using 5 N NaOH. No pH buffer was used. All samples were purged with N_2 gas for 30 – 120 min prior to transfer to an anaerobic chamber (97% N_2 and 3% H_2), where reactor solutions were prepared.

Text S3. Determination of the UV fluence rate

Incident fluence rate was used instead of the average fluence rate for experiments with a 222 nm KrCl* excimer lamp due to several practical and experimental reasons: 1) it is directly measurable at a specific distance from the lamp using a radiometer or actinometer, 2) reporting incident fluence rates facilitates easier comparison between studies and consistent reporting across experiments (222 nm vs. 254 nm), 3) at 222 nm, water and other constituents rapidly absorb UV light, leading to significant light attenuation. Using the incident fluence rate ensures the results are appropriately reflective of small reactor system, and 4) The KrCl* excimer lamp emits more collimated light compared to traditional UV lamp, making the incident fluence rate a more representative parameter for describing the UV intensity.

The incident UV fluence rate of the 222 nm KrCl* excimer lamp (Ushio, Care222) used in this study was determined using a potassium iodide-iodate ($KI/NaIO_3$) chemical actinometry, following the procedures outlined in a previous study.[2] The actinometer solution contained 0.6 M KI and 0.1 M $NaIO_3$ in a 0.01 M borate buffer (pH 9.2). The UV lamp was warmed up for 10 minutes to ensure stabilization. Approximately 17 mL of the actinometer solution was added to a quartz reactor (exterior diameter = 50 mm, and path length = 10 mm) covered with a polytetrafluoroethylene (PTFE) lid. The solution was placed under the lamp and mixed with a magnetic stirrer. Samples were taken at predetermined intervals over a 5-minute period, diluted (dilution factor =21), and the absorbance of triiodide (I_3^-) at 352 nm was measured using a Cary 100 UV/VIS spectrophotometer (Varian). The concentration of I_3^- was determined using a molar absorptivity of 26,400 $M^{-1}\times cm^{-1}$ at 352 nm. The incident UV fluence rate (E_0, mol photons$\cdot cm^{-2}\cdot s^{-1}$) was calculated using the following equation:

$$E_0 = \frac{(\Delta[I_3^-]/\Delta t)\times V_{reactor} \times U_\lambda}{\Phi \times A_{reactor}} \qquad \text{Eq. S1}$$

Where U_λ is the energy per mole photon at wavelength l (539 000 J per mol of photons at 222 nm), $A_{reactor}$ is the UV irradiation area of the reactor (cm^2) and $V_{reactor}$ is the quartz reactor volume (L), t is the irradiation time (s), Φ is the quantum yield of triiodide formation (0.94 at 222 nm). E_0 indicates the total UV photons at 222 nm entering the solution from the UV source. In this study, the estimated values ranged from 2.06E-9 to 3.63E-9 mol photons$\cdot cm^{-2}\cdot s^{-1}$ (1.11 to 1.96 mJ$\cdot s^{-1}\cdot cm^{-2}$) in Table S4.

The average incident fluence rate (mJ$\cdot s^{-1}\cdot cm^{-2}$) ($E_{0,ave}$) can be calculated using a water factor (WF) (Eq. S2)

$$E_{ave} = E_0 \times WF = \frac{E_0\times(1-10^{-al})}{a \times l\times \ln(10)} \qquad \text{Eq. S2}$$

where a (cm^{-1}) is the absorption coefficient (cm^{-1}), l (cm) is the effective path length. In the absence of sulfite, PFAS diluted solutions (25 μM) in deionized water showed low absorbance (0.035 ± 0.024) and a WF of 0.961 ± 0.027, resulting in $E_{0,ave}$ between 1.07 to 1.88 mJ$\cdot s^{-1}\cdot cm^{-2}$.

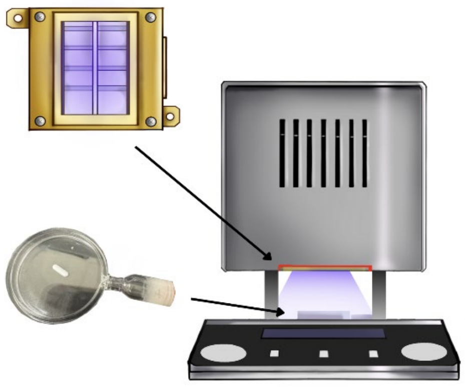

Figure S1. The illustration of the experimental setup.

Text S4. Fluoride measurements

The concentration of fluoride ions released during PFAS degradation was primarily determined by an ion-selective electrode (ISE) (Beckman Coulter 511141) connected to a Thermo Fisher Scientific Orion Versa Star Pro meter. Standard solution concentrations ranged from 0.002 mM to 2 mM (0.038- 38 mg/L). The slope of the potential (mV) versus log[F^-] (mg/L) ranged from 55 to 58 mV per log unit (Figure S2a). A 2 mL aliquot of the reaction sample was mixed with an equal volume of fluoride total ionic strength adjustor buffer solution (ISA) (HACH), and the F^- concentration was then measured using the ISE. The accuracy of F^- measurement by the ISE was validated by comparing ISE results with those obtained from ion chromatography for samples taken at all reaction times (Figure S2b).

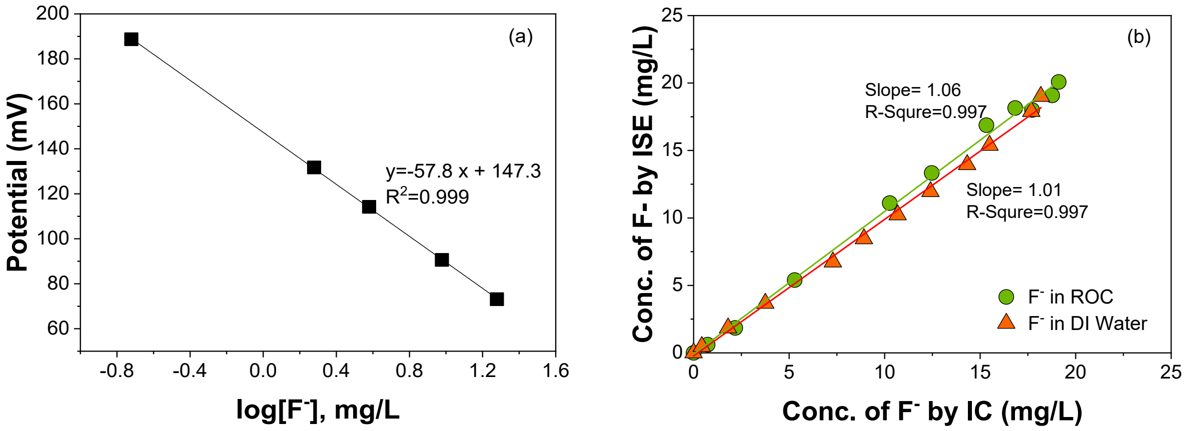

Figure S2. Calibration curve for ISE (a) and comparison of [F^-] between IC and ISE (b).

Text S5. Inorganic anion measurement

Anions, including fluoride, formate, chloride, nitrate, nitrite, bromide, bromate and chlorate, were analyzed using a Dionex Integrion and Aquion ion chromatography (ThermoFisher Scientific), equipped with Dionex IonPAc AS19 column (4 × 250 mm), a Dionex IonPac AG19 (4 × 50 mm) guard column, and a Dionex ADRS 600 (4 mm) suppressor. Anions were eluted with 10 mM KOH at a flow rate of 1.0 mL/min and a suppressor current of 50 mA. The column was maintained at 30 °C.

Text S6. Sulfite measurement

Sulfite concentrations were quantified by measuring the absorbance of thiol [3] at 412 nm following the reaction between sulfite and 5,5'dithiobis (2-nitrobenzoic acid) (DTNB).[4, 5] A 0.1 mL sample was withdrawn from the reactor and immediately diluted with ultrapure water at a 50:1 ratio. The dilution factor was adjusted according to the initial sulfite concentration. A 0.5 mL aliquot of the diluted sample was then added to a 4 mL of 100 mM phosphate (pH 7) and 0.5 mL of 2 mM DTNB solution. The samples were allowed to react for 5-10 minutes before measuring absorbance at 412 nm. Sulfite concentrations were calculated using the Beer-Lambert Law, with a molar absorptivity for thiol of 14000 $M^{-1} \times cm^{-1}$ at 412 nm.

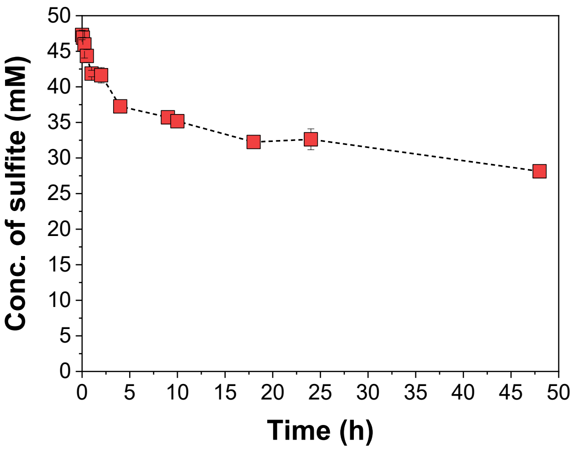

Figure S3. Sulfite concentrations during $PFAS_{mixture}$ decay in ROC by UV_{222}/sulfite-ARP. Conditions: $[PFAS_{mixture}]_0$ = 25 μM, $[Na_2SO_3]_0$ = 50 mM, N_2-purged, pH 12 (no buffer), and incident UV fluence rate = 3-3.63 × 10^{-9} mol photons·cm^{-2}·s^{-1}. Markers represent the mean of duplicate measurements, and error bars indicate the range between duplicates.

Text S7. PFAS measurements

Quality control check for native PFAS standards

Native standards of 50,000 ng/mL for perfluoro-n-octanoic acid (PFOA), perfluoro-n-butanoic acid (PFBA), sodium perfluoro-1-octanesulfonate (L-PFOS), and potassium perfluoro-1-butanesulfonate (L-PFBS) from Wellington laboratories LLC (Table S2) were mixed and diluted to create a working stock solution of 1500 ng/mL (PFOA + PFOS + PFBA + PFBS) using LC-MS grade methanol. This working stock solution (1,500 ng/mL) was further diluted to 22.5 ng/mL or 30 ng/mL in LC-MS grade methanol. The measured concentrations by LC-MS/MS are shown in Table S5.

Table S5. Recovery of four native PFAS mixture standards at 22.5 ng/mL

Sample	PFOA (ng/mL)	PFBA (ng/mL)	L-PFOS (ng/mL)	L-PFBS (ng/mL)
1st	20.46	22.59	22.52	21.54
2nd	20.23	23.32	22.81	21.57
3rd	20.69	22.80	21.99	22.06
Average	20.46	22.90	22.44	21.72
STDEV	0.23	0.37	0.42	0.30
Recovery (%)	91	102	100	97

The 3rd sample was prepared and analyzed on a different date than the 1st and 2nd samples.

PFAS measurement by LC-MS/MS

All PFAS measurements were performed with a triple quadrupole mass spectrometer (Altis, ThermoFisher Scientific, Waltham, MA) coupled to a binary pump HPLC (Vanquish, ThermoFisher Scientific). Mass spectrometer parameters were optimized for the PFAS under direct infusion at 5 $\mu L \cdot min^{-1}$ to identify the SRM transitions (precursor/product fragment ion pair) with the highest intensity. Samples were kept at 4°C on an autosampler before 10 µL were injected onto the instrument. A Hypersil Gold 5 µm, 50 x 3 mm column (ThermoFisher Scientific) at 30°C was used for chromatographic separation. A 9.5-minute gradient method was used with a solvent composed of 0.1% formic acid solution in water and a 0.1% formic acid solution in acetonitrile at a 0.6 $mL \cdot min^{-1}$ flow rate. TraceFinder 3.3 (ThermoFisher Scientific) was used for sample analysis and quantification. Final PFAS concentrations were normalized to the specific isotopically labeled PFAS surrogate concentration contained in each sample.

PFAS concentrations in direct photolysis experiments were diluted by a factor of 50-125 in a 0.1% NH_4OH in methanol, spiked with internal standard, injected into the LC-MS/MS system. PFAS concentrations in UV/sulfite-ARP experiments were first isolated from the background water matrix using solid phase extraction (SPE) followed by high performance injection into the

LC-MS/MS. Prior to SPE, samples were diluted (100:1) in ultra-pure water and spiked with a PFAS surrogate working stock (30 ng/mL). The PFAS surrogate working stock solution contained isotopically labeled standards for PFAS of interest. The SPE procedure employed a 16-port HyperSep Glass Block Vacuum Manifold (ThermoFisher Scientific) prepared with Oasis WAX 1cc cartridge, 30 mg, 30 μm (Waters) for SPE. Under low vacuum pressure (< 2 psi), up to 8 total Oasis WAX cartridges were conditioned three consecutive times using 1 mL of 0.1% NH_4OH in methanol followed by three consecutive times using 1 mL of 100% methanol. 1 mL of sample was then immediately added onto the Oasis WAX cartridge followed by 1 mL of 25 mM ammonium acetate to wash the cartridge. All cartridges were dried under low vacuum pressure (<2 psi) for 4-5 minutes. The vacuum manifold was then turned off, and cartridges were moved to the back of the vacuum manifold. Under low vacuum pressure (< 2 psi), 1 mL of 0.1% NH_4OH in methanol was added to elute PFAS off the cartridges into disposable, borosilicate glass collection tubes (VWR). The vacuum was then turned off, and all elution tubes were covered with parafilm before transferring the final product into Leap PAL Parts L-MARK vials. Lower limits of quantitation via this method (0.5 μg L^{-1}) are based on the lowest concentration used in the calibration curve (0.5 ng mL^{-1}), the volume of sample loaded onto the SPE cartridge (1 mL), and the volume of 0.1 % NH_4OH in methanol used to elute the cartridge (1 mL).

Estimation of initial $PFAS_{mixture}$ concentration spiked into the reactor

A $PFAS_{mixture}$ stock solution, containing PFBA, PFOA, PFBS, and PFOS was prepared at a concentration of 1 mM in ultrapure water. The solution was stirred and heated on a VWR hotplate stirrer until fully dissolved, then stored in a 250 mL-polypropylene bottle at room temperature. To accurately calculate the defluorination percentage (deF%), the initial $PFAS_{mixture}$ concentration, targeted at 0.025 mM (theoretical concentration) in a quartz reactor, was estimated before irradiation experiments by measuring the 1 mM PFAS stock solution. The 1 mM $PFAS_{mixture}$ stock solution prepared in ultrapure water was diluted to 0.1 μM using 0.1%(v/v) NH_4OH in methanol and then measured by LC-MS/MS. Table S6 presents the recovery of the 0.1 μM $PFAS_{mixture}$ diluted solution. Table S7 shows the $PFAS_{mixture}$ concentration in the reactor and the estimated fluorine concentration that could be released from the $PFAS_{mixture}$, based on the recoveries shown in Table S6. The total F^- concentrations released from $PFAS_{mixture}$ was calculated to be approximately 1.07 mM (Table S7), which was used to determine the defluorination percentage (%) in this study (Experiments 1-3 in Table S4). Table S8 presents the $PFAS_{mixture}$ concentration in the reactor and the fluorine concentration that could be released from the $PFAS_{mixture}$ for experiments 4 to 8 in Table S4.

Table S6. Recovery of 0.1 μM $PFAS_{mixture}$ diluted solution in experiments 1-3.

$PFAS_{mixture}$	Molecular weight (g/mol)	Measured conc. (ng/mL)	Measured conc. (mmol/L)	Recovery %
PFOA	436.052	33.29	0.076	76.3
PFBA	214.04	23.20	0.108	108.4
PFOS	538.22	46.63	0.087	86.6
PFBS	300.1	30.65	0.102	102.1

Table S7. Concentrations of the $PFAS_{mixture}$ and total F^- released in a reactor.

$PFAS_{mixture}$	Chemical Formula	Number of Fluorine atoms	PFAS conc. in a reactor (mM)	F^- released from PFAS (mM)
PFOA	$C_8HF_{15}O_2$	15	0.0190	0.2846
PFBA	$C_4HF_7O_2$	7	0.0269	0.1885
PFOS	$C_8HF_{17}O_3S$	17	0.0215	0.3660
PFBS	$C_4HF_9O_3S$	9	0.0254	0.2285

Total F^- concentration in the $PFAS_{mixture}$ for experiments 1-3 was estimated at 1.07mM.

Table S8. Recovery of 0.1 μM $PFAS_{mixture}$ diluted solution in experiments 4-8.

**Sample**	**PFOA** (mM)	**PFBA** (mM)	**PFOS** (mM)	**PFBS** (mM)
1st	0.076	0.076	0.085	0.109
2nd	0.074	0.080	0.082	0.110
Average=	0.075	0.078	0.083	0.109
STDEV	0.001	0.003	0.002	0.001
Recovery (%)	75.1	77.7	83.5	109.4

Total F^- concentration in $PFAS_{mixture}$ for experiments 4-8 was estimated at 1.01 mM.

Table S9. Recovery of 0.1 μM individual PFAS diluted solution in experiments 11-14.

**Parent compound**	**Surrogates**		
Recovery (%)	Avg. (ng/mL)	Recovery (%)	RSD (%)
PFOA 70.2 ± 0.9	28.9	M8PFOA 96.5	5.6
PFOS 59.7 ± 3.2	30.3	M8PFOS 101	3.1
PFBA 64.8 ± 1.1	31.6	M3PFBA 105.4	5.8
PFBS 94.9 ± 1.0	31.9	M3PFBS 106.4	8.5

Text S8. Individual PFAS degradation at pH 12.

The UV photolysis of individual PFBA, PFOA, PFBS, PFOS in deionized water at pH 12 under 222 nm irradiation was investigated (Figure S4). The results demonstrated near quantitative defluorination for each PFAS compound over the reaction time. The time-based first-order rate constants (k) followed the order: PFBS (0.026 h^{-1}) < PFOS (0.112 h^{-1}) < PFOA (0.952 h^{-1}) < PFBA (1.081 h^{-1}) (Table S10).

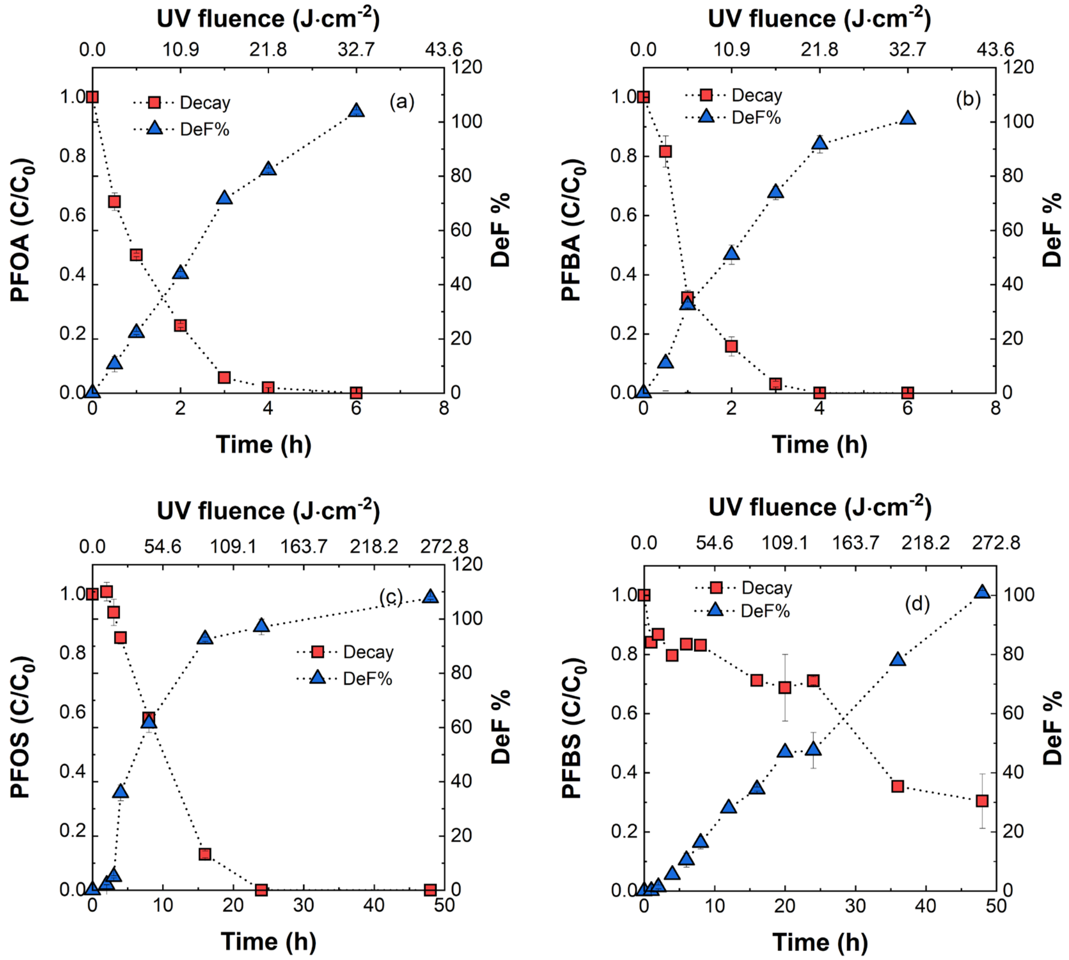

Figure S4. Photodegradation and defluorination percentages of PFOA (a), PFBA (b), PFOS (c), and PFBS (d) in deionized water at 222 nm. Conditions: $[PFBA]_0 = [PFOA]_0 = [PFBS]_0 = [PFOS]_0 = 25$ μM, N_2-purged, pH = 12, and incident UV fluence rate = 2.81×10^{-9} mol photons·cm^{-2}·s^{-1}. In Figure S4a-d, C_0 is the initial PFAS concentration measured at t = 0. Markers represent the mean of duplicate measurements, and error bars indicate the range between duplicates.

Table S10. Rate constant and defluorination efficiency of PFCAs.

PFCA[a] $C_nF_{2n+1}COO^-$	C_nF_{2n+1} chain length (n)	k_{obs} (h^{-1})			Fluence-based k (k_{obs_F}) ($cm^2 \cdot J^{-1}$)		
		pH 9, w/ N_2	pH 12, w/o N_2	pH 12, w/ N2	pH 9, w/ N_2	pH 12, w/o N_2	pH 12, w/ N_2
PFOA	N = 7	0.557	0.458	0.952 ± 0.003	0.139	0.115	0.175 ± 0.001
PFBA	N = 3	0.331	0.221	1.081 ± 0.051	0.059	0.055	0.198 ± 0.009

PFCA	F^- formation rate (R^{F^-}) (4 h), (mM/h)			deF(%) at t = 4 h			Fluence-based deF $\% \cdot cm^2 \cdot J^{-1}$		
	pH 9, w/ N_2	pH 12, w/o N_2	pH 12, w/ N_2	pH 9, w/ N_2	pH 12, w/o N_2	pH 12, w/ N_2	pH 9, w/ N_2	pH 12, w/o N_2	pH 12, w/ N_2
PFOA	0.019	0.028	0.062	27.5	41.3	87.5 ± 0.8	1.722	2.583	4.010 ± 0.034
PFBA	0.003	0.014	0.032	24.9	38.5	91.5 ± 3	1.101	2.406	4.194 ± 0.139

[a] Conditions: individual PFCA conc. = 25 μM, and at 20 °C. Estimated UV fluence rates for all experiments are listed in Table S4.

Energy Efficiency Analysis

EE/O ($kWh \cdot m^{-3}$ $order^{-1}$), defined as the energy needed to reduce the contaminant concentration by one order of magnitude in treating 1 m^3 of water,[6] was calculated for PFAS defluorination in UV_{222}-only and UV_{222}/sulfite conditions. EE/O is calculated via eq. (S3). The observed pseudo-first-order rate constant for PFAS degradation under UV_{222}-only and UV_{222}/sulfite conditions are denoted as k_{obs} (h^{-1}) (Table S10) and $k_{obs,SO_3^{2-}}$ (Figure S14), respectively. Power is the lamp output power (0.011 kW) for the KrCl* excimer lamp. Volume (V) is the treated volume (~17 × 10^{-6} m^3), and α is the wall plug efficiency of the lamps (10% for the KrCl* lamp). As shown in Table S11, under UV_{222}-only conditions, removal of dissolved oxygen and an increase in pH reduced the electrical energy per order (EE/O) by factors of 2.1 and 1.7, respectively for PFOA, and by 4.9 and 3.3, respectively, for PFBA. In Figure S5, the EE/O values for degrading the four individual PFAS compounds for the UV_{222}/sulfite ARP are 13-25-fold lower than those for the UV_{222}-only condition.

$$\mathrm{EE/O} = \frac{2.303 \times \mathrm{power}}{k_{\mathrm{obs,UV}}\left(or\ k_{obs,SO_3^{2-}}\right) \times \mathrm{V} \times \alpha} \qquad \text{Eq. S3}$$

Table S11. Electrical energies per order for PFAS degradation by UV-only and UV/sulfite ARPs.

	UV_{222}-only		UV_{222}/sulfite ARP	
	Matrix	EE/O × 10^{-3} ($kWh \cdot m^{-3}$)	Matrix	EE/O × 10^{-3} ($kWh \cdot m^{-3}$)
PFOA	pH 9 w/ N_2	26.75		
	pH 12 w/o N_2	32.53		
	pH 12 w/ N_2	15.65 ± 0.05	10 mM sulfite at pH 12 w/ N_2	1.09
PFBA	pH 9 w/ N_2	45.02		
	pH 12 w/o N_2	67.43		
	pH 12 w/ N_2	13.79 ± 0.65	10 mM sulfite at pH 12 w/ N_2	1.05
PFOS	pH 12 w/ N_2	133.05	10 mM sulfite at pH 12 w/ N_2	5.39
PFBS	pH 12 w/ N_2	573.14	10 mM sulfite at pH 12 w/ N_2	38.31

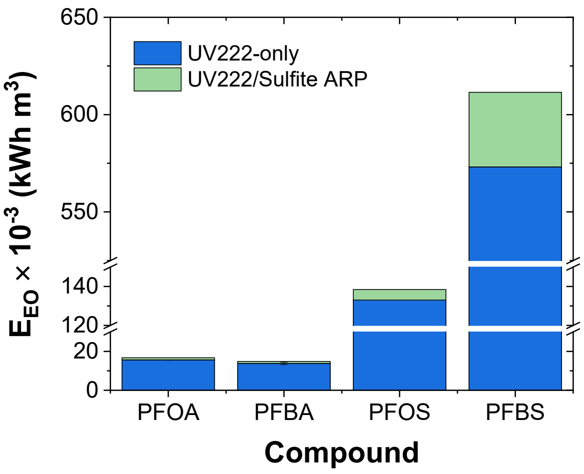

Figure S5. Electrical energy per order (EE/O) for PFAS degradation by UV_{222}-only and UV_{222}/sulfite ARP. Conditions: $[PFAS]_0 = 25$ μM, $[Na_2SO_3]_0 = 10$ mM, N_2-purged, and $pH_0 \approx 12$. The corresponding rate constants are presented in Table S10 and Figure S14. The lamp output power is 11 W for the KrCl* excimer lamp.

Determination of scavenging capacity

Under conditions where dissolved oxygen is present or the pH is moderate, the first-order scavenging capacity (k'_S)[5] was calculated assuming that the primary scavengers of e^-_{aq} are PFAS, protons (H^+), and dissolved oxygen (O_2). The scavenging capacity (k'_S) was calculated as:

$$k'_S = \sum k_{Si,e^-_{aq}}[S_i] \qquad \text{Eq. S4}$$

Where $k_{Si,e^-_{aq}}$ is the bimolecular rate constant for reaction with e^-_{aq}(M^{-1} s^{-1}), and $[S_i]$ is the concentration of the scavenger (M). The relevant reactions and rate constants are:

$$H^+ + e^-_{aq} \rightarrow H^{\cdot} \quad k \approx 2.3 \times 10^{10}\ (M^{-1}\ s^{-1})^{7} \qquad \text{Eq. S5}$$

$$O_2 + e^-_{aq} \rightarrow O_2^{\cdot-} \quad k \approx 1.9 \times 10^{10}\ (M^{-1}\ s^{-1})^{7} \qquad \text{Eq. S6}$$

At pH 12 without N_2 purging (air-saturated), the k'_S increased by nearly two orders of magnitude rising from 1.2×10^5 s^{-1} to 5.2×10^6 s^{-1}. This significant increase suggests that O_2 acts as a primary scavenger of e^-_{aq}. In contrast, under N_2-purged conditions, k'_S remained constant at 1.2×10^5 s^{-1} as the pH decreased from 12 to 9, indicating that protons do not significantly scavenge e^-_{aq} within this pH range. For these calculations, $[H^+]$ was derived from the solution pH. Dissolved oxygen concentrations for air-saturated conditions were estimated using Henry's law constant for O_2 ($k_H = 1.3 \times 10^{-3}$ mol·L^{-1}·atm^{-1} at 21°C, ref. https://www.henrys-law.org), while the residual O_2 in N_2-purged condition was estimated at 0.2 mgL^{-1}.[8]

Table S12 presents the calculated decadic absorption coefficient and corresponding water factors for PFAS solution in the presence of dissolved oxygen and at moderate pH. The result indicates

that hydroxide is the primary contributor to light screening, whereas dissolved oxygen has a minor effect. At pH 12, the absorption coefficient is approximately 0.17 cm^{-1}, resulting in a WF of ~ 0.82. Comparison of N_2-purged and air-saturated conditions at pH 12 shows oxygen has a negligible impact on light screening. At pH 9, the water factor approaches unity, indicating minimal light screening.

Table S12. Calculated absorption coefficient (a) and water factor (WF) for PFAS solution.

Condition	PFOA		PFBA		PFOS		PFBS	
	[1]$a = \sum \varepsilon_i C_i$	WF	$a = \sum \varepsilon_i C_i$	WF	$a = \sum \varepsilon_i C_i$	WF	$a = \sum \varepsilon_i C_i$	WF
(i) pH12 + N_2	0.169	0.825	0.168	0.826	0.167	0.827	0.167	0.827
(ii) pH12 + No N_2	0.175	0.820	0.173	0.821	0.173	0.822	0.173	0.822
(iii) pH9 + N_2	0.003	0.996	0.003	0.996	0.001	0.998	0.001	0.999

[1]a = absorption coefficient of the solution at 222 nm ($=\sum \varepsilon_i C_i$, cm^{-1}). The molar absorptivities are provided in Table S17. Concentrations are 25 μM for PFAS, 10 mM for OH^-, 8.7 mg/L for air-saturated conditions, and 0.2 mg/L for N_2-saturated conditions. The water factor was calculated using Eq. S2.

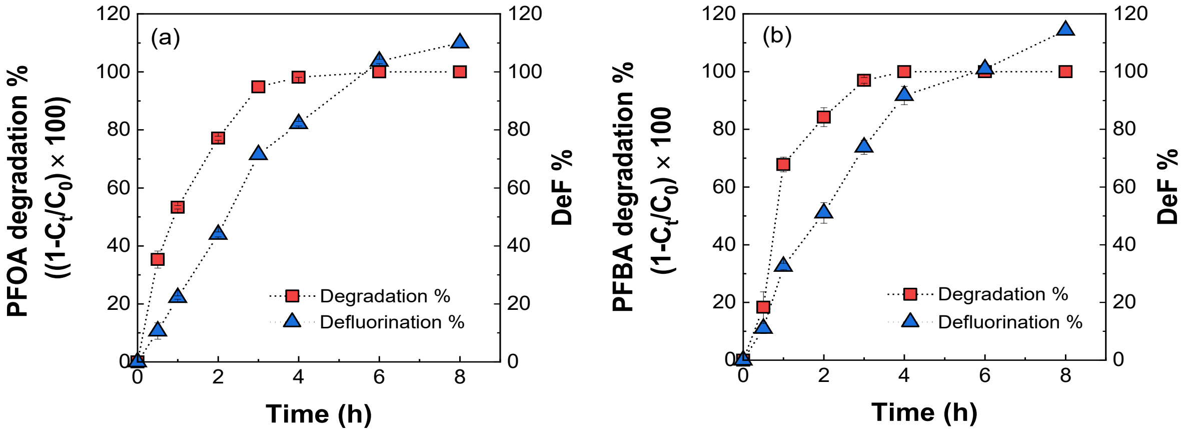

Figure S6. The percentages of degradation and defluorination of PFCAs under 222 nm irradiation. Conditions: $[PFBA]_0 = [PFOA]_0 = 25$ μM, pH = 12, N_2-purged, and 222 nm irradiance.

Dark control experiments

Degradation tests for PFOA and PFOS in the dark showed negligible defluorination over 4 h, and 48 h, respectively. MCAA degradation in the presence of sulfite under dark showed no dechlorination.

Table S13. Experimental conditions for light control

	Light- control (PFOA)[1]	Light-control (PFOS)[2]
Initial F^- conc.	0.0006 mM at t = 0 h	0.004 mM at t = 0 h
F^- conc. At time t	0.0006 mM at t = 4 h	0.003 mM at t = 48 h

[1]conditions: $[PFOA]_0 = 0.02$ mM, pH 7 (1mM phosphate), N_2-purged, and UV fluence rate = 3.6×10^{-9} mol photons cm^{-2} s^{-1} and [2]$[PFOS]_0 = 25$ μM, pH 12 (no buffer), N_2- purged, and UV fluence rate = 3×10^{-9} mol photons cm^{-2} s^{-1}. All fluoride concentrations were determined by ion chromatography.

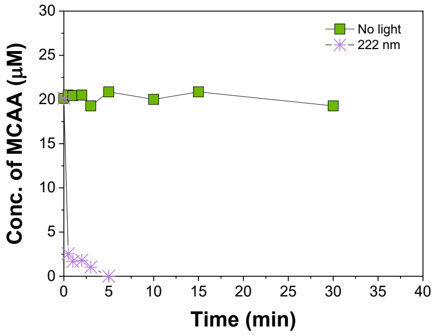

Figure S7. Degradation of monochloroacetic acid under 222 nm. Conditions: $[MCAA]_0 = 20\ \mu M$, $[Na_2SO_3]_0 = 0.5$ mM, pH 9.7 - 9.8 (1mM borate buffer), and incident UV fluence rate = $3.59 \times 10^{-9} \pm 3.45 \times 10^{-10}$ mol photons·cm^2·s^{-1}.

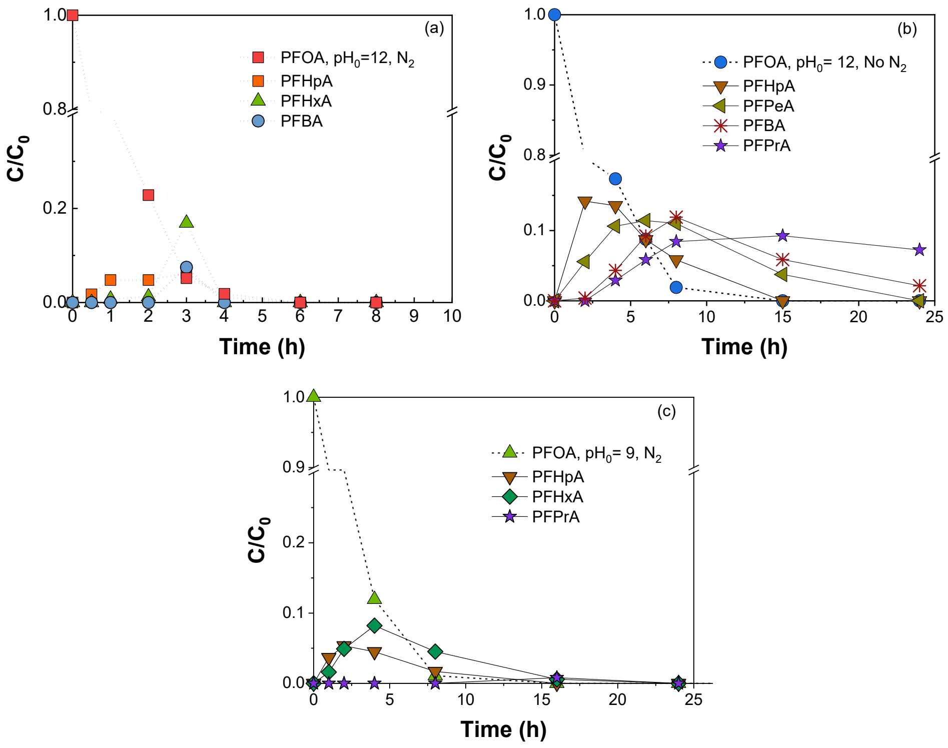

Figure S8. Degradation products of PFOA by UV_{222} photolysis. Conditions: $[PFOA]_0 = 25\ \mu M$, pH 12, and 222 nm irradiance. The sample was diluted 100-fold prior to analysis by LC-MS/MS. C_0 is the initial measured concentration of a parent compound (PFOA). The limit of detection (LOD) is 50 ng/mL for PFBA, PFOA, PFBS, PFOS, perfluoroheptanoic acid (PFHpA) and perfluorohexanoic acid (PFHxA).

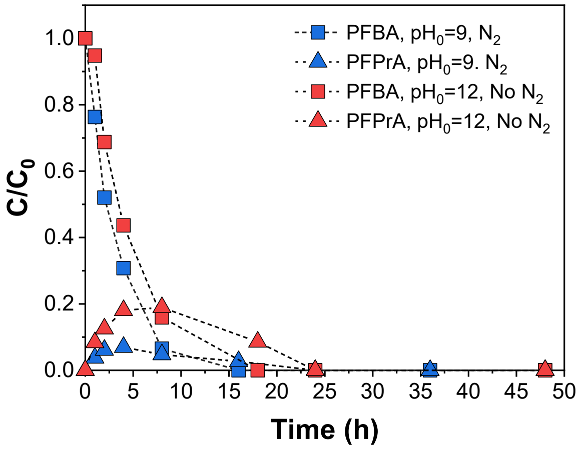

Figure S9. PFBA degradation products by UV_{222} photolysis. Conditions: $[PFBA]_0$ = 25 µM, and 222 nm irradiance. C_0 is the initial concentration of PFBA.

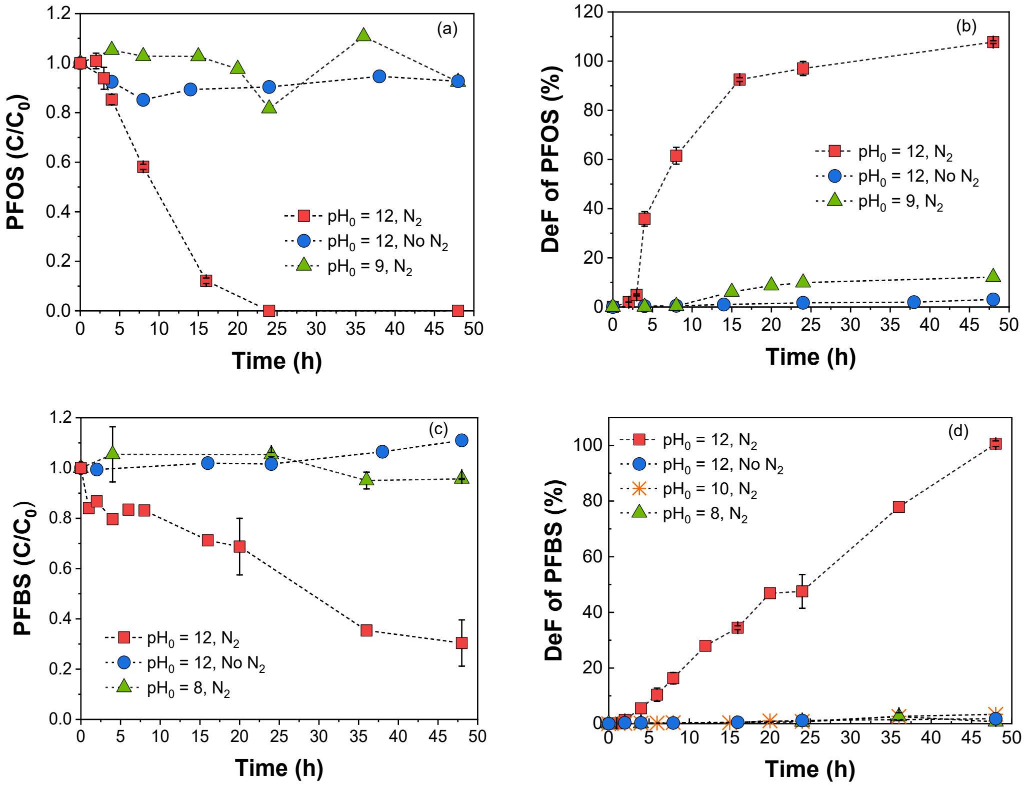

Figure S10. Degradation and defluorination of PFOS and PFBS. Conditions: $[PFOS]_0$ = $[PFBS]_0$ = 25 µM, initial pH = 12 or 8 –10. No buffer was used in pH control. C_0 is the initial concentration of a parent PFAS.

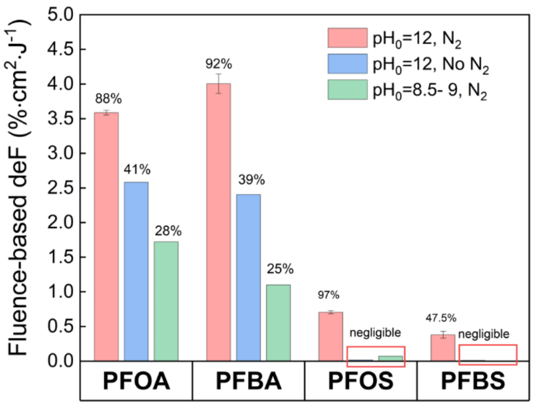

Figure S11. Fluence-normalized defluorination for four individual PFAS compounds at 222 nm dependent on the solution pH and N_2 saturation. Conditions: $[PFAS]_0 = 25$ μM, $pH_0 = 12$, and 222 nm irradiation. Fluence-based deF% was estimated at t = 4 h for PFOA and PFBA and at t = 24 h for PFOS and PFBS. The numbers on the top of column present the defluorination percentage of PFCA (at t = 4 h) and PFSA (at t = 24 h).

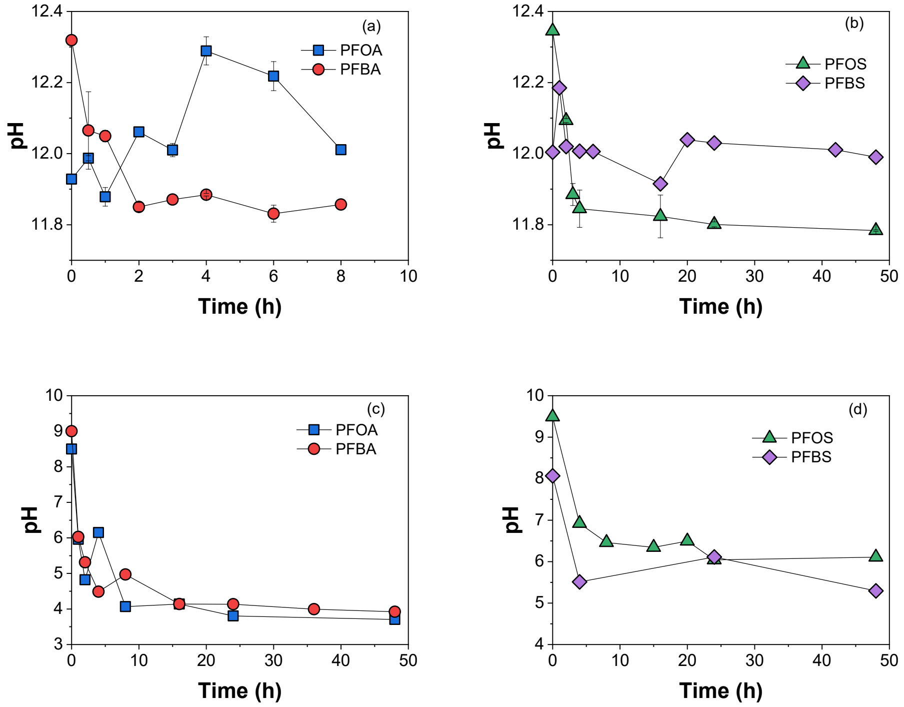

Figure S12. Solution pH changes during degradation of individual PFAS compounds under UV-only conditions. Conditions: The initial pH was adjusted to 12 or 8-9 using 5 N NaOH, and no buffer was used for pH control.

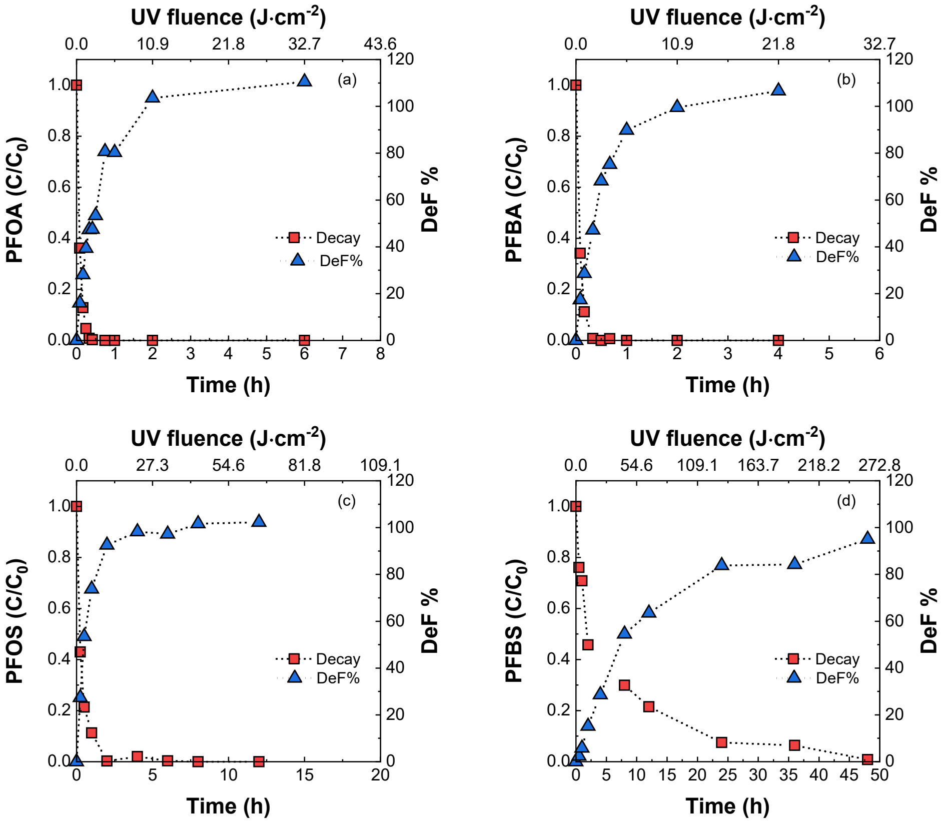

Figure S13. Degradation and defluorination of PFAS parent compounds by UV_{222}/sulfite ARP. Conditions: $[PFAS]_0 = 25$ μM, $[Na_2SO_3]_0 = 10$ mM, N_2-purged, $pH_0 = 12$ (no buffer), 20 °C and incident UV fluence rate = 2.81 ×10^{-9} mol photons·cm^{-2}·s^{-1}.

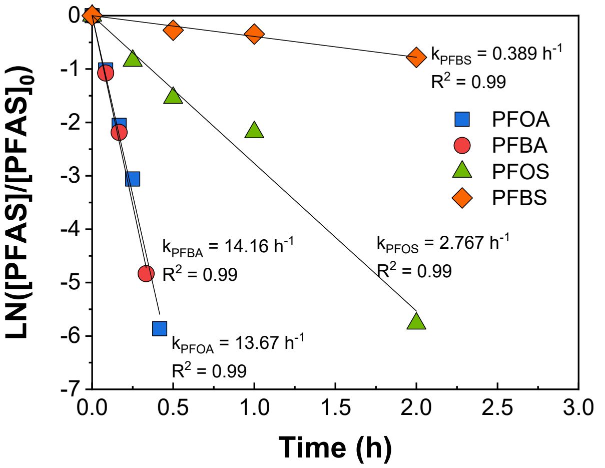

Figure S14. Kinetics of PFAS degradation in the UV_{222}/sulfite ARP. Conditions: $[PFAS]_0 = 25$ μM, $[Na_2SO_3]_0 = 10$ mM, N_2-purged, $pH_0 = 12$ (no buffer), 20 °C and incident UV fluence rate = 2.81 ×10^{-9} mol photons·cm^{-2}·s^{-1}.

Text S9. Behavior of PFAS degradation in a mixture solution

While previous studies have predominantly focused on the reactivity of individual PFAS, evaluating their behavior in complex mixtures is critical, as they coexist in impacted water matrices. In this study, the pseudo-first-order rate constant (k_{obs}) for all target PFAS decreased markedly in a four-PFAS mixtures compared to single-compound systems, both in the absence and presence of sulfite (Table S14, Figures S15 and S16).

Table S14. Comparison of time-based rate constants of single and mixed PFAS.

compound	The pseudo-first-order rate constant (h^{-1})			
	A single PFAS compound		PFAS in a four-PFAS mixture	
	No sulfite	10 mM sulfite	No sulfite	50 mM sulfite
PFOA	0.9517	13.672	0.7658	6.073
PFBA	1.0812	14.162	0.4482	3.415
PFOS	0.1115	2.767	0.0465	0.341
PFBS	0.0255	0.39	0.0019	0.065

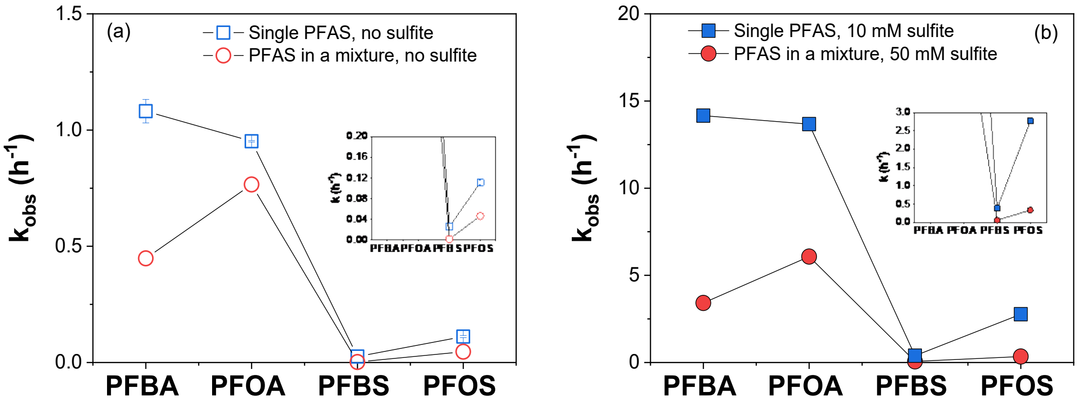

Figure S15. Time-based rate constants of single vs. mixed PFAS by UV only (a) and UV_{222}/sulfite ARP (b). Conditions: initial single PFAS concentration and each PFAS concentration in a mixture is 25 μM, and pH 12.

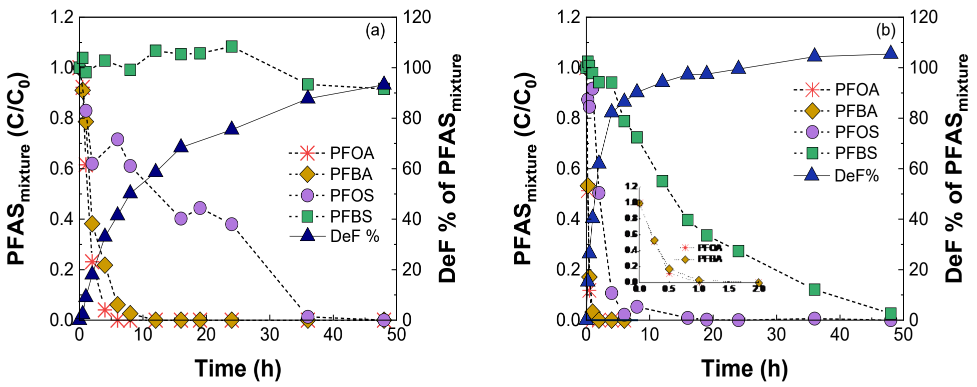

Figure S16. Degradation and defluorination of $PFAS_{mixture}$ under UV_{222}-only (a) and UV_{222}/sulfite ARP (b) in deionized water. Conditions: $[PFAS_{mixture}]_0$ = 25 μM, $[Na_2SO_3]_0$ = 0 (a) or 50 mM (b), N_2-purged, and pH 12. Incident UV fluence rate = 3 $\times 10^{-9}$ mol photons·cm^{-2}·s^{-1}.

Text S10. Quantum yield calculations for PFAS UV photolysis

The general form of the equation used to describe pseudo first-order direct photolysis reactions under monochromatic irradiation at wavelength λ is given by

$$\frac{dC_i}{dt} = -\Phi \frac{I_{0\lambda}}{z}\left(1 - 10^{-(\alpha_\lambda + \varepsilon_\lambda C_i)\ell}\right)F_i \qquad \text{Eq. S7}$$

where C_i is the concentration of the chemical of interest i in mol/L, t is time, Φ is the quantum yield, $I_{0\lambda}$ is the photon irradiance in mmol photons/cm^2/s, z is the depth of the water in cm, α_λ is the decadic absorbance of the solution (excluding the chemical of interest i), ε_λ is the molar absorptivity of the chemical of interest in M^{-1} cm^{-1}, ℓ is the pathlength of light in the water in cm, F_i is the fraction of light absorbed by the chemical of interest is given by $\varepsilon_\lambda C_i/(\varepsilon_\lambda C_i + \alpha_\lambda)$. In our experimental system, the effective path length by H_2O_2 method[5, 9, 10] was estimated at 1.02 cm and we assumed that the water depth (z) and the pathlength of light in the water (ℓ) are equivalent because there is no light scattering by particles.

UV photolysis quantum yields of PFAS were calculated using this equation (S7), which for single compound solutions of each PFAS and considering the above $z = \ell$ assumption simplifies to

$$\frac{dC_i}{dt} = -\Phi \frac{I_{0\lambda}}{\ell}\left(1 - 10^{-(\varepsilon_\lambda C_i)\ell}\right) \qquad \text{Eq. S8}$$

where dC_i/dt is chosen to be the initial rate of PFAS degradation and is calculated as $k_{PFAS}[PFAS]_0$ where k_{PFAS} is the first order photodegradation rate constant and $[PFAS]_0$ is the initial concentration.

Since the PFAS degradation at pH 12 under UV_{222} only conditions involve mechanisms beyond direct photolysis-most notably hydroxide-mediated pathways-we use the term "apparent quantum yield (Φ_{app})" in the main manuscript to describe their photon-normalized degradation efficiency at pH 12.

Table S15. Literature pK_a values, and measured molar absorptivity, rate constants, and quantum yields.

Compound	ε ($M^{-1}\cdot cm^{-1}$) [a]	k_{obs} (h^{-1})[b]	$\Phi_{app,PFAS}$[c]	$\Phi_{app,F-}$	Φ_{PFAS}[11] (at pH 8.5)	pK_a
PFOA	132.2	0.952 ± 0.003	0.374	1.684	0.137	2.3-3.4[12, 13]
PFBA	68.3	1.081 ± 0.051	0.822	1.595	0.116	0.24-0.4[12, 13]
PFOS	46.7	0.112	0.124	1.333	n.r.	-3.3 - +3.2[14, 15]
PFBS	34.9	0.026	0.036	0.230	n.r.	< -1.85[16]

[a]This value was estimated using the measured concentration of an individual PFAS stock solution in deionized water (no pH control and no deaerated). The apparent quantum yield was calculated using Eq. S9-10. [c]The time-based pseudo-first-order rate constant (k_{obs}, h^{-1}) for PFAS degradation by UV_{222} photolysis at pH 12. Initial fluoride formation rates ($R_0^{F^-}$) are 0.0829, 0.0422, 0.026, 0.0047 mM/h for PFOA, PFBA, PFOS, and PFBS, respectively (Figure S17).

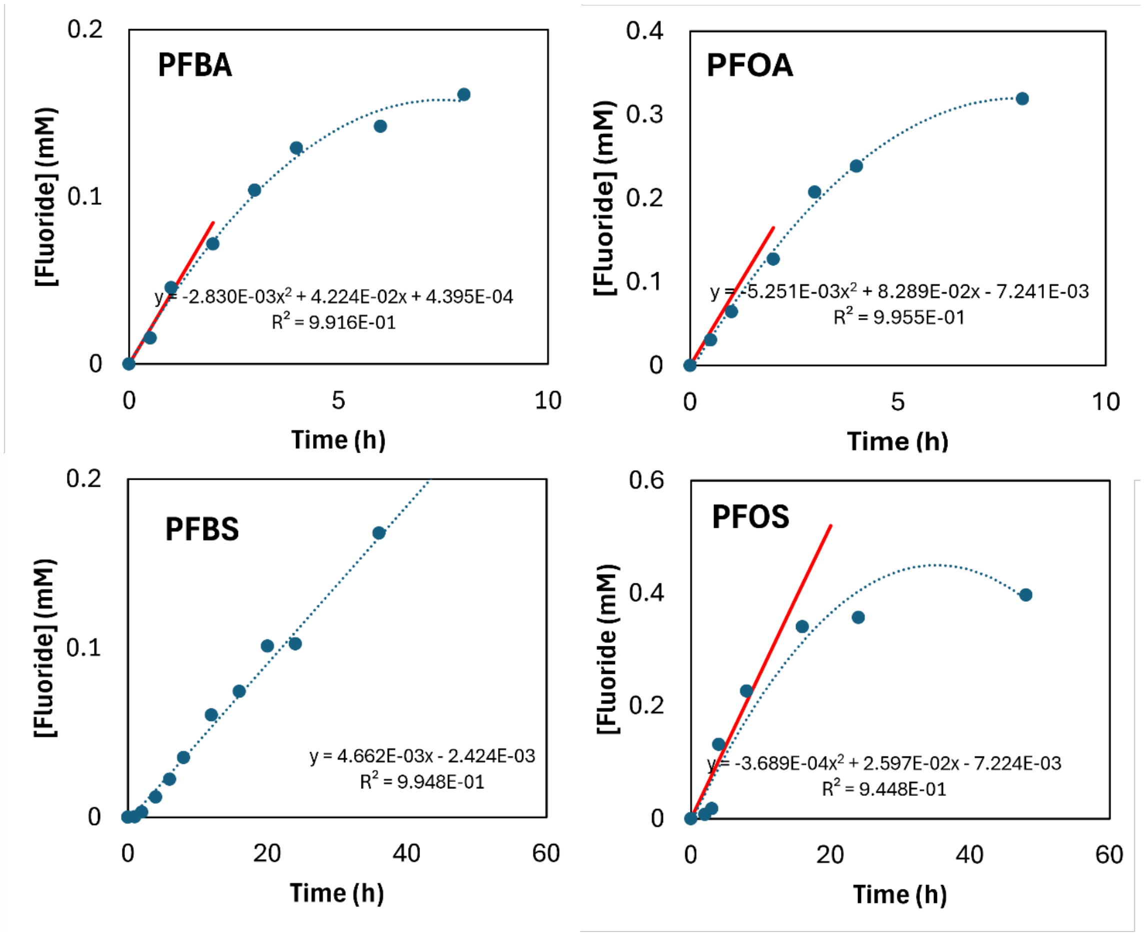

Figure S17. [F⁻] versus time during UV_{222} photolysis of individual PFAS compounds. Condition: For PFOA, PFBA, and PFOS, initial rates of fluoride formation were calculated from the derivative evaluated at time zero (red line) of a quadratic model fit to the data (blue dashed line). For PFBS, the rate of fluoride formation was determined from a linear model (dashed line).

Apparent quantum yield corrected by light absorption of OH^-

The apparent quantum yields ($\Phi_{app,PFAS}$) of target PFAS presented in the manuscript were estimated at pH 12 under UV_{222}-only conditions using Eq. (S9), yielding values of 0.036 (PFBS), 0.124 (PFOS), 0.374 (PFOA), and 0.822 (PFBA). The observed degradation of PFCAs was attributed to both direct photolysis and hydrated electron-mediated reduction, with the latter driven by hydroxide photolysis at pH 12. In contrast, PFSAs were primarily degraded by the latter pathway. At pH 12, the high concentration of OH^- contributes significantly to the solution absorbance at 222 nm. Given its non-negligible molar absorptivity ($\varepsilon = 16.6\ M^{-1}\cdot cm^{-1}$ at 222 nm), the standard quantum yield calculation (Eq. (S11)[6, 17]) was modified to account for hydroxide light absorption. Accounting for OH^- absorption at 222 nm, the corrected apparent quantum yields ($\Phi_{app,PFAS,corr}$) using Eq. (S11) were 0.0002 (PFBS), 0.0008 (PFOS), 0.0057 (PFOA), and 0.0068 (PFBA). Under N_2-purged conditions, the trace dissolved oxygen concentration (~ 0.2 mg/L)[8] had a negligible

effect on light absorption and was not included in calculating $\Phi_{app,PFAS}$ or $\Phi_{app,PFAS,corr}$. The $\Phi_{app,PFAS}$ is normalized to photons absorbed by PFAS only, whereas $\Phi_{app,PFAS,corr}$ is normalized to the total photons absorbed by the solution, which are predominantly absorbed by OH^-. Therefore, $\Phi_{app,PFAS,corr}$ is substantially lower than the $\Phi_{app,PFAS}$.

$$\Phi_{app,PFAS} = \frac{k_{obs,PFAS}[PFAS]_0}{\frac{E_0}{\ell}\left(1-10^{-(\varepsilon_{PFAS}[PFAS]_0+\varepsilon_{OH^-}[OH^-])\ell}\right)\times\left(\frac{\varepsilon_{PFAS}[PFAS]_0}{\varepsilon_{PFAS}[PFAS]_0+\varepsilon_{OH^-}[OH^-]}\right)} \quad \text{Eq. S9}$$

$$\Phi_{app,F^-} = \frac{R_0^{F^-}}{\frac{E_0}{\ell}\left(1-10^{-(\varepsilon_{PFAS}[PFAS]_0+\varepsilon_{OH^-}[OH^-])\ell}\right)\times\left(\frac{\varepsilon_{PFAS}[PFAS]_0}{\varepsilon_{PFAS}[PFAS]_0+\varepsilon_{OH^-}[OH^-]]}\right)} \quad \text{Eq. S10}$$

$$\Phi_{app,PFAS,corr} = \frac{k_{obs,PFAS}[PFAS]_0}{\frac{E_0}{\ell}\left(1-10^{-(\varepsilon_{PFAS}[PFAS]_0+\varepsilon_{OH^-}[OH^-])\ell}\right)} \quad \text{Eq. S11}$$

$$\Phi_{app,F^-,corr} = \frac{R_0^{F^-}}{\frac{E_0}{\ell}\left(1-10^{-(\varepsilon_{PFAS}[PFAS]_0+\varepsilon_{OH^-}[OH^-])\ell}\right)} \quad \text{Eq. S12}$$

where $k_{obs,PFAS}$ is the PFAS pseudo-first-order photodegradation rate constant (s^{-1}) (Table S10), $[PFAS]_0$ is the measured initial PFAS concentration ($mol\cdot L^{-1}$), E_0 is incident fluence rate at 222 nm (mol photons cm^{-2} s^{-1}), ℓ is the effective path length (cm), and ε_{PFAS} and ε_{OH^-} is the molar absorptivity of the PFAS and OH^- at 222 nm ($M^{-1}\cdot cm^{-1}$) (in Table S15 and S17), and $R_0^{F^-}$ is the initial rate of fluoride ion formation ($M\cdot s^{-1}$). The concentration of OH^- is 10 mM (pH 12).

Table S16. Apparent quantum yield corrected by OH^- light absorption

	$\Phi_{app,PFAS,corr}$	$\Phi_{app,F^-,corr}$
Compound	By Eq. (S11)	By Eq. (S12)
PFOA	0.0057	0.026
PFBA	0.0068	0.013
PFOS	0.0008	0.008
PFBS	0.0002	0.001

Text S11. Reproducibility of $PFAS_{mixture}$ defluorination experiments

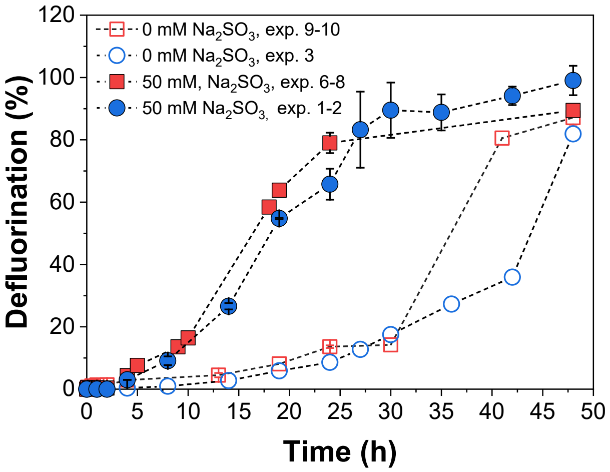

Figure S18. Defluorination of the $PFAS_{mixture}$ in ROC by UV_{222}–only and UV_{222}/sulfite ARP. Conditions: $[PFAS_{mixture}]_0 = 25$ µM, $[Na_2SO_3]_0 = 50$ mM, N_2-purged, and pH 12. Marker represents the mean of duplicate or triplicate measurements, and error bars indicate the range between replicates. Detailed experimental conditions are in Table S4.

Table S17. Molar absorptivity and quantum yield of water matrix components at 222 and 254 nm.

Inorganic ions	ε ($M^{-1}\cdot cm^{-1}$)	ε ($M^{-1}\cdot cm^{-1}$)	$\Phi_{e_{aq}^-}$	$\Phi_{e_{aq}^-}$
	222 nm	254 nm	222 nm	254 nm
SO_3^{2-}	1110[a]	18.7[a], 18.14[18], 18.4[19]	0.123[20]	0.116[19]
NO_3^-	2540[a], 2500[21], 2747[22]	3.3[a], 4.7[23], 3[22]		0.17[21]
NO_2^-	3440[a], 3500[21], 3507[22]	12.6[a], 12[22]		
CO_3^{2-}	42.3[23]	negligible[23]		
Cl^-	negligible[23]	negligible[23]		
Br^-	77.6[23]	negligible[23]		
I^-	11500[a], 11527[22]	190[a], 220[24], 272[22]	0.27±0.05[25]	
Sulfate	negligible[23]	negligible[23]		
MCAA	51[a]	negligible[23]		
OH^-	16.6 [a]	negligible[23]		
O_2	21.2[26]			

[a]This value was measured in this work.

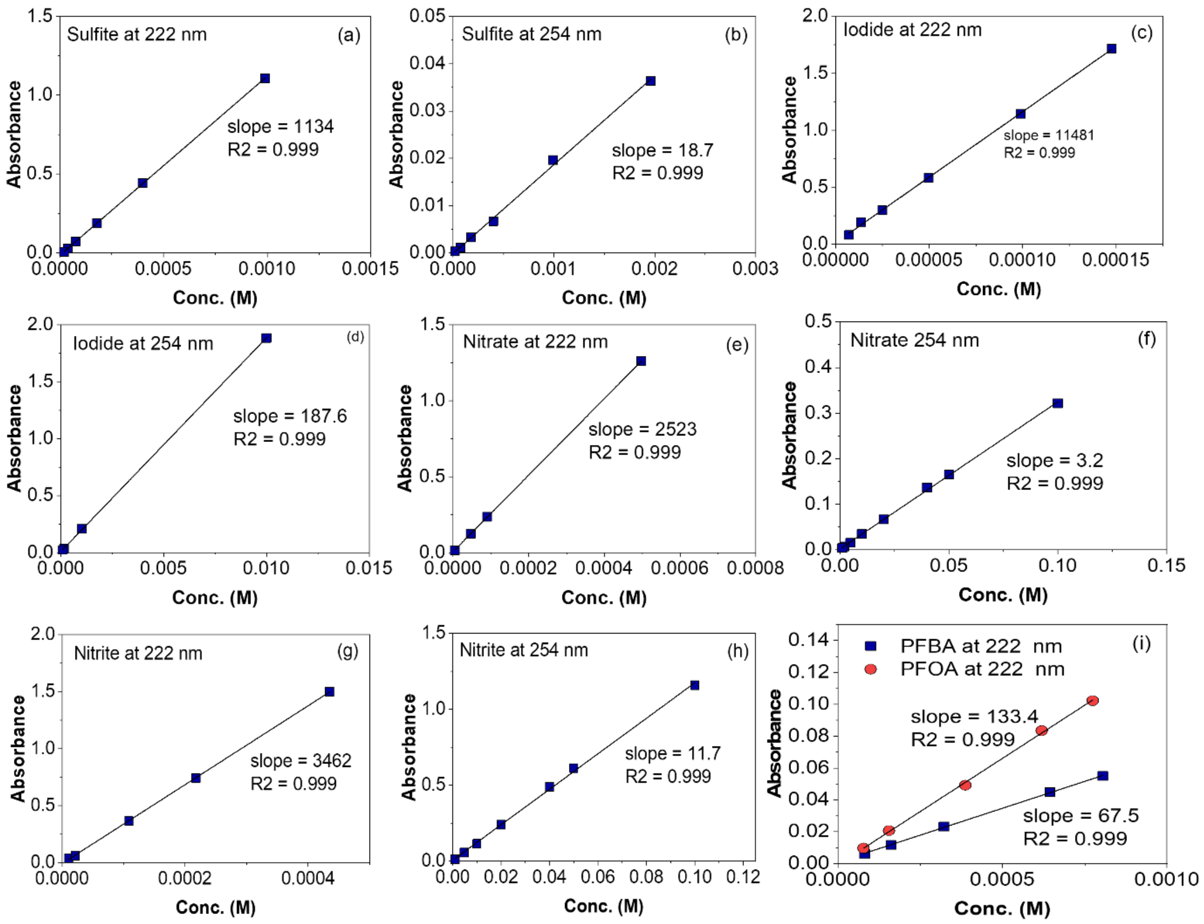

Figure S19. Molar absorptivity of sulfite, iodide, nitrate, and nitrite, and PFAS at 222 and 254 nm.

Text S12. Mechanistic evidence for hydrated electron generation from OH^- photolysis

I) Mechanistic evidence from MCAA degradation at different pH values.

The pK_a of MCAA is approximately 2.86, [27] therefore, it remains fully deprotonated across the entire investigated range (pH 7-12). As shown in Table S18, the degradation rates under 222 nm at pH 7 (0.0224 min^{-1}) and pH 9.8 (0.021 min^{-1}) are nearly identical and we assume that the rate constant at pH 7 is attributed to direct photolysis (k_{dp}). However, at pH 12, the pseudo-first order rate constant (k_{obs,OH^-}) increases dramatically to approximately 0.157-0.263 min^{-1}. This significant enhancement at pH 12 provides strong evidence for the formation of e_{aq}^- from OH- photolysis (OH- + hv → •OH + e_{aq}^-).[28] This is further supported by the increase in chloride recovery, which rose from ~ 50% at lower pH to 67% at pH 12, indicating an accelerated reductive dechlorination.

Determination of apparent quantum yield (Φ_{app})

To quantify the e_{aq}^- quantum yield from OH^- photolysis, we assumed that MCAA degradation at pH 12 is the sum of direct photolysis and indirect photochemical reactions mediated by e_{aq}^- generated from hydroxide and/or sulfite.

i) Under UV-only conditions:

$$k_{obs,OH^-} = k_{dp} + k_{e_{aq}^-,OH^-} \qquad \text{Eq. S13}$$

ii) Under UV-sulfite conditions:

$$k_{obs,SO_3^{2-}} = k_{dp} + k_{e_{aq}^-,OH^-} + k_{e_{aq}^-,SO_3^{2-}} \qquad \text{Eq. S14}$$

where k_{obs,OH^-} is the pseudo-first-order rate constant for MCAA degradation at pH 12 under 222 nm irradiation without sulfite, k_{dp} is the rate constant for direct photolysis of MCAA (measured at pH 7 under 222 nm), and $k_{e_{aq}^-,OH^-}$ represents the contribution from e_{aq}^- generated from hydroxide photolysis. The $k_{obs,SO_3^{2-}}$ is the pseudo-first-order rate constant for MCAA degradation in the presence of sulfite at pH 12, and $k_{e_{aq}^-,SO_3^{2-}}$ represents the contribution from e_{aq}^- generated from sulfite photolysis.

The apparent quantum yield (Φ_{app,e_{aq}^-}) of hydrated electrons generated from hydroxide based on MCAA decay were calculated using the Eq. S15. [19] This Φ_{app,e_{aq}^-} is defined as the ratio of the moles of e_{aq}^- formed from hydroxide photolysis to the moles of photons absorbed by OH^-, with the assumption that MCAA is the only scavenger of hydrated electrons in this system, neglecting reactions with H^+ due to high pH and O_2 under N_2-purged conditions (scavenging capacity of

MCAA is 2-8 × 10^4 s^{-1} vs. scavenging by trace O_2 and H^+ combined at pH 12/deaeration is 1.2 ×10^5 s^{-1}). The residual DO concentration under N_2-purged was estimated at 0.2 mg L^{-1}. [8]

$$\Phi_{app,e_{aq}^-} = \frac{r_0\left(=k_{e_{aq}^-,OH^-} \times C_{MCAA}\right)V}{I_0\left(1-10^{-(\varepsilon_{OH}C_{OH}+\varepsilon_{MCAA}C_{MCAA})l}\right)\times\frac{\varepsilon_{OH}C_{OH}}{\varepsilon_{OH}C_{OH}+\varepsilon_{MCAA}C_{MCAA}}} \quad \text{Eq. S15}$$

where r_0 is the formation rate of e_{aq}^- from hydroxide (OH^- + hv → •OH + e_{aq}^-) (M s^{-1}), I_0, the photon flux determined by actinometry (mol photons s^{-1}), ε is the molar absorptivity at 222 nm (ε_{MCAA} = 51 M^{-1} cm^{-1}; ε_{OH^-} = 16.6 $M^{-1}cm^{-1}$ in Table S17). The absorbance at 222 nm of 6.75 mM NaOH solution was approximately 0.112. The effective path length (*l*, cm) is 1.02 cm, and C_{OH} and C_{MCAA} are the initial concentration of NaOH and MCAA.

Our calculated quantum yield (Φ_{app,e_{aq}^-}) for e_{aq}^- generated from OH^- at pH 12 and 222 nm irradiation was 0.158 ± 0.014. Published data reported Φ ~ 0.112 from OH^- at 193 nm.[29, 30] Li et al. reported a quantum yield Φ = 0.116 ± 0.002 for e_{aq}^- from sulfite photolysis at 254 nm, determined via the MCAA degradation at pH 10.1.[19]

Table S18. Experimental conditions of MCAA degradation in the absence of sulfite under 222 nm.

Exp.	pH	$[MCAA]_0$	k_{obs}	Recovery of chloride ($[Cl^-]_{at\ t\_final}$ / $[MCAA]_0$) × 100	Apparent Quantum yield (Φ_{app,e_{aq}^-})
M1	7	0.021 mM	0.0224 min^{-1}	49 %	
M2	9.8	0.022 mM	0.021 min^{-1}	55%	
M3	9.8	0.044 mM	0.041 min^{-1}	49%	0.012
M4	12	0.048 mM	0.263 min^{-1}	67 %	0.168
M5	12	0.080 mM	0.163 min^{-1}	N.A.	0.164
M6	12	0.072 mM	0.157min^{-1}	N.A.	0.141

The initial MCAA concentration was experimentally measured. Incident UV fluence rate was 3.59 × 10^{-9} ± 3.45 × 10^{-10} mol photons cm^{-2} s^{-1}. The effective path length was 1.02 cm, and the reactor volume was 17.3 mL. Solutions were N_2-purged for 1 h. Solutions at pH 7 contained 1 mM phosphate buffer, while those at pH 9.8 and pH 12 contained 1 mM borate buffer. The pH was adjusted as needed using 5 N NaOH.

Quantification of hydrated electrons

Hydrated electrons (e_{aq}^-) were quantified based on MCAA degradation under two conditions: i) UV_{222}-only at pH 12, where e_{aq}^- is generated from OH^- photolysis, and ii) UV_{222}/sulfite at pH 12 where e_{aq}^- is generated from both sulfite and OH^-. At steady state, the concentration of hydrated electrons ($[e_{aq}^-]_{ss}$) is defined between the formation rate and decay rates of e_{aq}^- ; Formation rate of e_{aq}^- = Decay rate of e_{aq}^-. Assuming that MCAA is the only significant scavenger of e_{aq}^-, the steady-state concentration of hydrated electrons can be simplified as [6]:

i) UV-only conditions (pH 12, without sulfite)

$$\left[e_{aq}^{-}\right]_{ss,UV} = \frac{(k_{obs,OH^-} - k_{dp,pH7})}{k'_{MCAA}} \qquad \text{Eq. S16}$$

ii) UV/sulfite conditions (pH 12, with sulfite)

$$\left[e_{aq}^{-}\right]_{ss,UV/SO3} = \frac{(k_{obs,SO_3^{2-}} - k_{dp,pH7})}{k'_{MCAA}} \qquad \text{Eq. S17}$$

The $\left[e_{aq}^{-}\right]_{ss}$ was calculated to be (2.87 ± 0.099) × 10^{-12} M and 1.08 × 10^{-11}M for the UV_{222}-only and UV_{222}/sulfite conditions, respectively, at pH 12 in N_2-purged deionized water under 1.95 mJ s^{-1} cm^{-2}. Here, the bimolecular rate constant of MCAA with e_{aq}^{-} (k'_{MCAA}) is 1.0 × 10^9 M^{-1}·s^{-1}.[5] The $k_{obs,SO_3^{2-}}$ was determined to be 0.673 min^{-1} at pH 12, with initial concentrations of 0.045 mM MCAA and 5 mM sulfite. Under UV/sulfite conditions at pH 12, hydrated electrons responsible for MCAA degradation are predominantly generated from sulfite photolysis (73%), with a contribution from hydroxide photolysis (27 %).

Yin et al.[6] reported the rate constant of MCAA degradation (k_{dp}) at pH 7 under 222 nm irradiation as (5.2 ± 0.14) × 10^{-5} (s^{-1}), along with $k_{obs,SO_3^{2-}}$ of (8.31 ± 0.009) × 10^{-4} (s^{-1}) and a steady state hydrated electron concentration ($\left[e_{aq}^{-}\right]_{ss}$) of (7.79 ± 0.0063) × 10^{-13} M . These values were obtained under the conditions: $[sulfite]_0$ = 1 mM, $[MCAA]_0$ = 0.01 mM, [DO] < 0.3 mg L^{-1}, and incident fluence rate = 0.43 mJ s^{-1} cm^{-2}. Also, we compared the value of $\left[e_{aq}^{-}\right]_{ss}/E_0$, $\left[e_{aq}^{-}\right]_{ss}$ normalized by the incident fluence rate, with published data by Yin et al.[6] at pH 7.

Table S19. Comparison of $[e_{aq}^{-}]_{ss}/E_0$ for MCAA degradation with literature values.

Wavelength	Sulfite (mM)	pH_0	$\left[e_{aq}^{-}\right]_{ss}/E_0$ (M·cm^2·s·mJ^{-1})	Ref.
222nm	0	12	(1.47 ± 0.051) ×10^{-12}	From this study
222nm	5	12	5.54 ×10^{-12}	From this study
222nm	1	7	(1.44 ± 0.0012) ×10^{-12}	[6]
254nm	1	7	(2.98 ± 0.09) × 10^{-14}	[6]

Table S20. Calculation of EE/O for MCAA degradation and comparison with literature data.

	This work		Literature data[6]	
Wavelength	222 nm	222 nm	222 nm	254nm
pH_0	12	12	7	7
Sulfite (mM)	0	5	1	1
$[MCAA]_0$ (mM)	0.045	0.045	0.01	0.01
k_{obs} (h^{-1})	15.78	40.38	~ 2.99	~ 0.058
the wall-plug efficiency (α)	0.1	0.1	0.1	0.3
Solution volume (mL)	17	17	50	50
Lamp output (W)	11	11	15	9
EE/O (kWh·m^3)	944	369	~ 2309[6]	~ 23798[6]

II) Mechanistic evidence from methanol spiking

To investigate the role of e^-_{aq} in PFAS defluorination at high pH under 222 nm irradiation, methanol was employed as a hydroxyl radical ($^{\bullet}$OH) scavenger. Photolysis of OH^- at 222 nm is proposed to generate both $^{\bullet}$OH and e^-_{aq}. In the absence of scavenger (e.g., methanol), the reactivity of e^-_{aq} is limited by rapid recombination with $^{\bullet}$OH ($k \approx 3\times10^{10}$ $M^{-1}\cdot s^{-1}$) [7] (Eq. S18), which serves as a primary sink for the generated electrons. Addition of methanol suppresses this recombination (Eq. (S19)), thereby increasing the steady-state concentration of e^-_{aq} for reductive PFAS degradation.

The enhancement in defluorination upon methanol addition was more pronounced for PFOS than that for PFOA (Figure S20), indicating that PFSAs degradation is more strongly dependent on e^-_{aq} availability. This is consistent with the fact that PFSAs are not readily degraded via true direct photolysis and rely primarily on e^-_{aq} mediated pathways, whereas PFCAs can undergo (in)direct photolysis at 222nm. Furthermore, the characteristic in induction period "lag phase" observed during PFOS and PFBS defluorination under UV-only conditions or in complex water matrices was eliminated upon methanol addition. This result suggests that the lag phase is governed by competition for e^-_{aq} by background scavengers, and $^{\bullet}$OH quenching by methanol enhances e^-_{aq} availability, particularly impacting PFSA degradation.

$$e^-_{aq} + {}^{\bullet}\mathrm{OH} \rightarrow OH^- \quad k \approx 3\times10^{10}\ \mathrm{M^{-1}\ s^{-1}} \qquad \text{Eq. S18}^{7}$$

$$CH_3OH + {}^{\bullet}\mathrm{OH} \rightarrow {}^{\bullet}CH_2OH + H_2O \quad k \approx 1.9\times10^{9}\ \mathrm{M^{-1}\ s^{-1}} \qquad \text{Eq. S19}^{7}$$

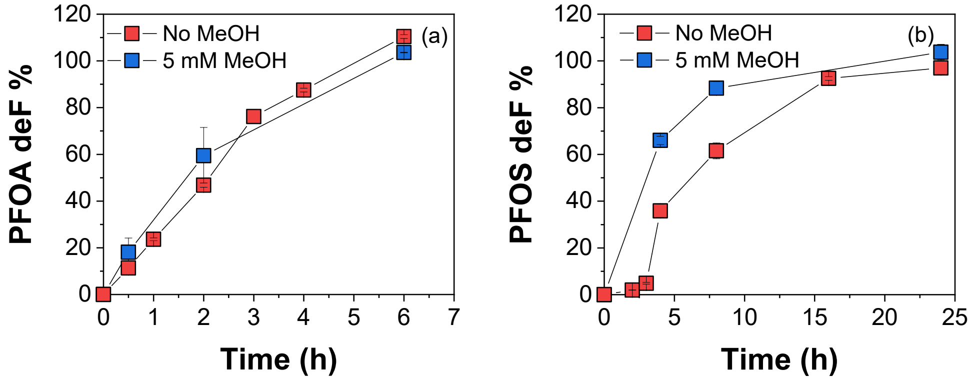

Figure S20. Effect of addition of 5 mM methanol on PFAS defluorination. Conditions: $[PFAS]_0$ = 25 µM, N_2-purged, pH_0 = 12 (10 mM NaOH), and incident fluence rate = 3.3×10^{-9} mol photons $cm^2\cdot s^{-1}$. All fluoride concentrations were measured by ion selective electrode (ISE).

Text S13. Estimation of light absorption fraction of major constituents in ROC

The light absorption of different species was calculated using $A_i = \varepsilon_i C_i$, where ε_i is molar absorptivity and C_i is the concentration measured for each specie. The absorbance of dissolved organic matter was estimated by subtracting the sum of A_i values from the total absorbance of the diluted water sample, yielding a $\varepsilon_{222,DOM}$ of 5.15 L $mg_C^{-1}\cdot m^{-1}$ = 618 L $mol_C^{-1}\cdot cm^{-1}$, which is in good agreement with values from prior publications.[23] The fraction of incident light absorbed by each species was then calculated as A_i/A_{total}, where A_{total} is the total absorbance of the diluted water sample.

In this revision, we calculate $\varepsilon_{222,DOM}$ directly according to the reviewer's suggestion and arrive at a value of 5.15 L $mg_C^{-1}\cdot m^{-1}$ = 618 L $mol_C^{-1}\cdot cm^{-1}$, which is in good agreement with expectations. We stand by the original light fraction calculations as being correct. The table below details these calculations, and we have added them (Table S21). In the UV/sulfite ARP at 222 nm, DOM is indeed a minor fraction of the light absorption mostly due to the high concentration of the sulfite e_{aq}^- source. Please note in the below calculations that the total absorbance of the ROC at 222 nm (A_{222}) was measured on a 121-fold diluted sample; therefore, the light absorption calculations are also presented at this 121-fold dilution:

Table S21. Light absorption calculations on 121-fold diluted ROC

**Calculations**	**F_i (%)**
$A_{222,sulfite}$ = (0.047 mol L^{-1})÷121× (1110 L mol^{-1} cm^{-1}) = 0.431 cm^{-1}	83 %
$A_{222,nitrate}$ = (0.0025 mol L^{-1}) ÷121× (2540 L mol^{-1} cm^{-1}) = 0.0525 cm^{-1}	10.1 %
$A_{222,nitrite}$ = (0.0005 mol L^{-1}) ÷121× (3440 L mol^{-1} cm^{-1}) = 0.0142 cm^{-1}	2.5 %
$A_{222,sulfite}+A_{222,nitrate}+A_{222,nitrite}$ = 0.4977 cm^{-1}	95.6 %
**$A_{222,ROC}$ = 0.519 cm^{-1} *total ROC absorbance at 222 nm***	
The difference of (0.519 – 0.4977) cm^{-1} is attributable to DOM	
$A_{222,DOM}$ = (0.519 – 0.4977) cm^{-1} = 0.0213 cm^{-1}	4.4 %
**$\varepsilon_{222,DOM}$ = 0.0213 cm^{-1} ÷ (52.1 mg_C L^{-1} ÷ 121) = 0.0515 L mg_C^{-1} cm^{-1}**	
**$\varepsilon_{222,DOM}$ = 0.0515 L mg_C^{-1} cm^{-1} = 5.15 L mg_C^{-1} m^{-1} = 618 L mol_C^{-1} cm^{-1}**	

Table S22. Calculated light absorption fraction of major constituents in ROC

Compound	Conc.	ε_{222nm}	Fraction of light absorption at 222 nm (%)	ε_{254nm}	Fraction of light absorption at 254 nm (%)
Nitrate	2.5 mM	2540 $M^{-1}\cdot cm^{-1}$	10.1 ± 0.2	4.7 $M^{-1}\cdot cm^{-1}$	0.62 ± 0.08
Nitrite	0.5 mM	3440 $M^{-1}\cdot cm^{-1}$	2.5 ± 0.03	12.6 $M^{-1}\cdot cm^{-1}$	0.31 ± 0.03
Sulfite	47 mM	1110 $M^{-1}\cdot cm^{-1}$	83.0 ± 2.7	18.7 $M^{-1}\cdot cm^{-1}$	46.6 ± 3.3
DOM[a]	52.1 mg_C L^{-1}	618 L $mol_C^{-1}\cdot cm^{-1}$	4.4 ± 2.6	225 L $mol_C^{-1}\cdot cm^{-1}$	52.4 ± 3.4

[a]The total absorbance of the ROC sample was measured after diluting (dilution factor = 121). Nitrate and nitrite concentrations were determined by IC (Figure S23), and sulfite concentration (Figure S3) was measured using the DTNB colorimetric method (Text S6). The absorbance of DOM was estimated by

subtracting the sum of the absorbances of nitrate, nitrite, and sulfite ($\sum\varepsilon_i C_i$) from the total absorbance of ROC sample.

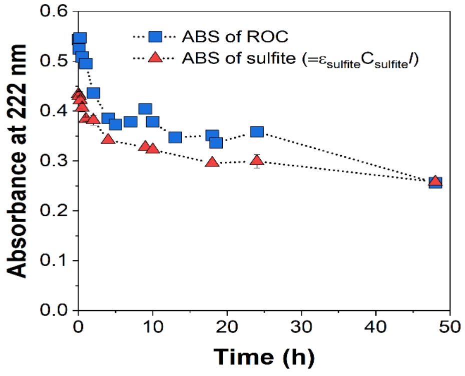

Figure S21. Total absorbance and estimated absorbance from sulfite concentrations for 121-fold diluted ROC at 222 nm.

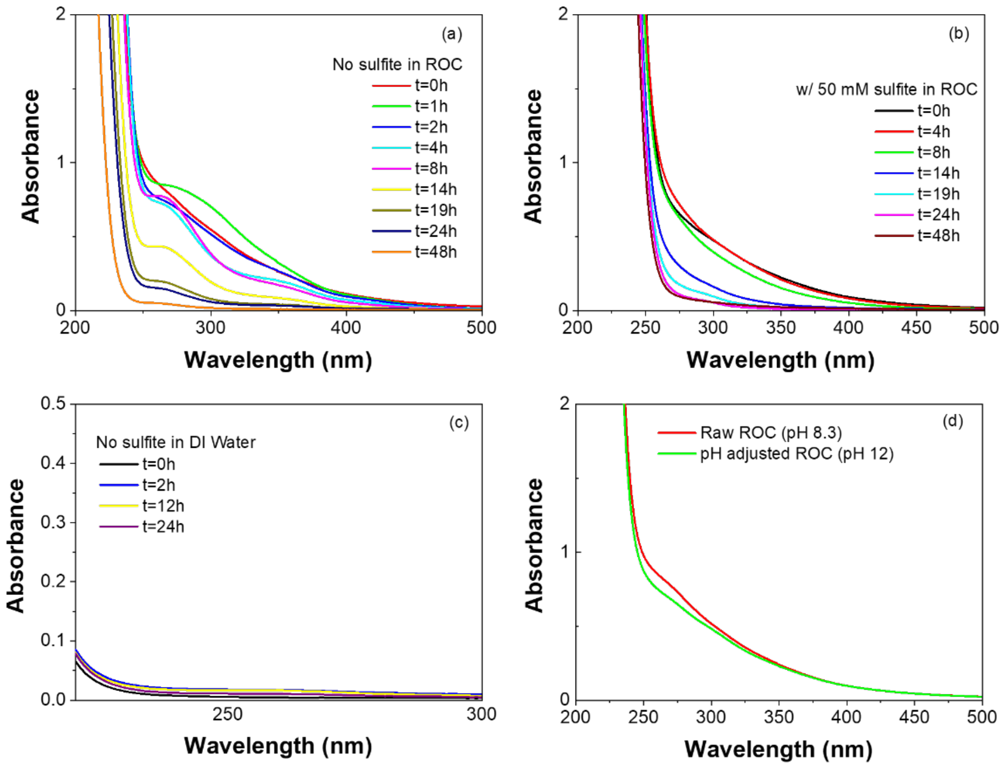

Figure S22. UV-visible spectra in deionized water and ROC samples. Conditions: $[PFAS_{mixture}]_0 =$ 25 μM, pH 12, and 222 nm irradiation. Conditions: (a) $[Na_2SO_3]_0 =$ 0 mM in ROC; (b) $[Na_2SO_3]_0$ = 50 mM in ROC; (c) $[Na_2SO_3]_0 =$ 0 mM in DI water; and (d) raw ROC samples without $PFAS_{mixture}$. Detailed experimental conditions are described in Table S4.

Nitrate and nitrite decay in ROC under 222 nm irradiation

Previous studies of KrCl* lamps in water treatment have demonstrated the potential of reaction byproducts driven by the additional photochemistry activated by 222 nm light (e.g., nitro compounds), which would impart toxicity in the product water. These issues are likely exacerbated in our ROC matrix, which contains 36 mgN $L^{-}1$ nitrate and 52 mgC L^{-1} DOM. Thus, the benefits of enhanced PFAS destruction at 222 nm must be balanced with the formation of toxic byproducts generated by interactions between DOM and reactive nitrogen species. Product water toxicity is a major hurdle for employing KrCl* lamps in water treatment which requires a comprehensive evaluation. Although speculative, it is possible that in our UV/sulfite-ARP many of these byproducts could be degraded due to the high fluence and high reactivity of putative byproducts (e.g., nitro compounds)[31,32] with e_{aq}^{-}. Figure S23 presents the nitrate and nitrite decay in ROC by UV_{222} – only and UV_{222}/sulfite-ARP.

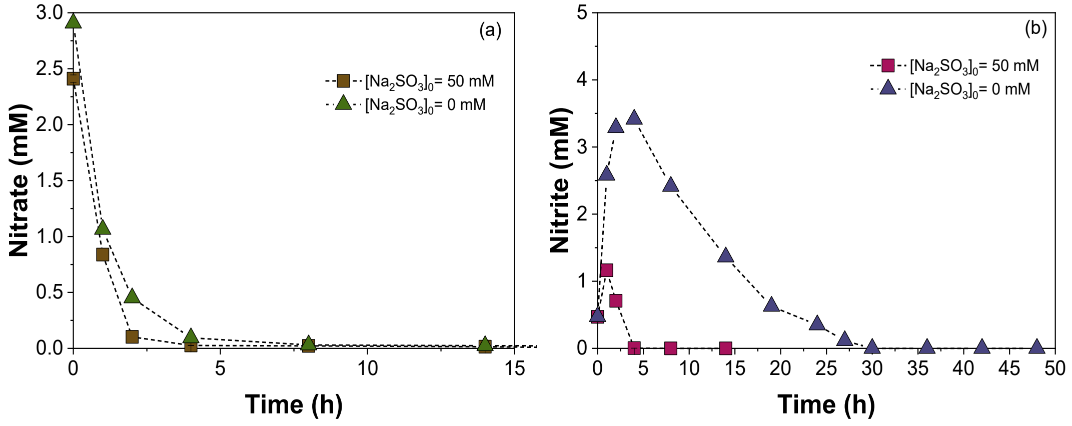

Figure S23. Nitrate (a) and nitrite (b) profiles during $PFAS_{mixture}$ decay in ROC. Conditions: $[PFAS_{mixture}]_0 = 25$ μM, N_2-purged, and pH 12 (exp. 1-3 in Table S4).

Text S14. Quantum yield for nitrate decay and nitrite formation from nitrate at 222 nm

The quantum yields for nitrate decay and nitrite formation from nitrate were estimated based on nitrate direct photolysis in deionized water at 222 nm monochromatic source. Experimental conditions are summarized in Table S23 and Table S24.

Table S23. Rate constants and quantum yields for nitrate decay by UV_{222} photolysis.

Exp. No	$[nitrate]_0$ (M)	pH	Media	k_{obs} (min^{-1})	$\varepsilon_{nitrate} \times C_{0,nitrate} \times L$	I_0 (mol photons cm^{-2} s^{-1})	$\Phi_{nitrate}$
N1	5.00E-03	12	DI Water	0.019	1.27E+01	4.57E-09	0.35
N2	5.00E-05	12	DI Water	0.476	1.27E-01	4.57E-09	0.34
N3	2.00E-05	10	DI Water	0.572	5.08E-02	4.57E-09	0.38

$\varepsilon_{nitrate}$ is the molar absorptivity of nitrate at 222 nm (2540 $M^{-1} \cdot cm^{-1}$), L is the path length (cm).

The quantum yield (Φ_{nitrate}) was calculated using the following equation:

$$\Phi_{\text{nitrate}} = \frac{k_{obs,nitrate} \cdot C_{0,nitrate}}{\frac{I_0}{L} \cdot \left(1 - 10^{-\varepsilon_{nitrate} C_{0,nitrate} L}\right)} \qquad \text{Eq. S20}$$

where $k_{obs,\ nitrate}$ is the observed rate constant for nitrate decay, $C_{0,nitrate}$ is the initial nitrate concentration, I_0 is the incident fluence rate at 222 nm measured by iodide-iodate actinometry (mol photons cm^{-2} s^{-1}), $\varepsilon_{nitrate}$ is molar absorptivity of nitrate at 222 nm.

Table S24. Initial rates and quantum yield of nitrite formation from nitrate at 222 nm.

Exp. No	$r_{0,nitrite}$ (mM/s)	$\Phi_{nitrite}$ (mole Ein^{-1})
N1	1.017	0.22
N2	0.286	0.25
N3	0.212	0.42

The quantum yield of nitrite formation from nitrate decay was calculated using the equation

$$\Phi_{\text{nitrite}} = \frac{r_{nitrite}}{\frac{I_0}{L} \cdot \left(1 - 10^{-e_{nitrate} C_{0,nitrate} L}\right)} \qquad \text{Eq. S21}$$

where $r_{0,nitrite}$ is the initial formation rate of nitrite, determined as the concentration of nitrite formed over 30 min (exp. N1), 3 min (exp. N2), 1 min (exp. N3) in Figure S24, showing a linear increase over time.

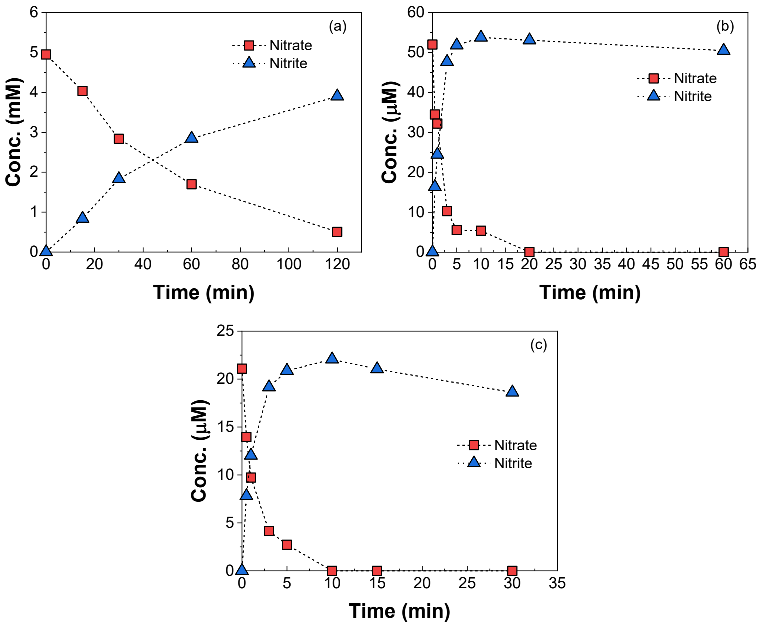

Figure S24. Nitrate decay and nitrite formation by UV222 photolysis. Conditions: (a) $[Nitrate]_0$ = 5mM (exp. N1), pH 12 (1 mM borate buffer), (b) $[Nitrate]_0$ = 50 μM (exp. N2), pH 12 (1mM borate buffer), and (c) $[Nitrate]_0$ =20 μM (exp. N3), pH 10 (1mM borate buffer). Incident UV fluence rate is 4.57 ×10^{-9} mol photons·cm^{-2}·s^{-1}.

The nitrate photolysis quantum yield results are consistent with wavelength-dependent quantum yields for nitrate photolysis. Goldstein and Rabani [33] found that the quantum yield for nitrate photolysis increased as the excitation wavelength decreased. At 220 nm, the quantum yields were $\Phi = 0.092$ for the reaction $NO_3^- + hv \rightarrow {}^{\bullet}NO_2 + O^{\bullet -}$ (Reaction 1), and $\Phi = 0.284$ for $NO_3^- + hv \rightarrow ONOO^-$ (Reaction 2). At 254 nm, $\Phi = 0.037$ and $\Phi = 0.102$ for Reactions 1 and 2, respectively.[33] Mack and Bolten reported $\Phi = 0.17$ for nitrite formation at 254 nm. [21] In this work, Φ for nitrate decay in deionized water by UV_{222} photolysis was estimated to be 0.35 ± 0.02, while Φ for nitrite formation from nitrate ranged from 0.02 to 0.42, depending on initial nitrate concentration ($[NO_3^-]_0$ = 0.02~5 mM, pH 10-12).

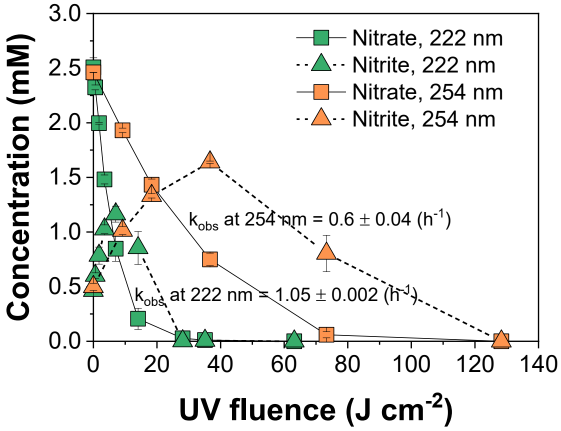

Figure S25. Nitrate and nitrite profiles at different wavelengths (222 and 254 nm) during $PFAS_{mixture}$ treatment in ROC. Conditions: $[PFAS_{mixture}]_0$ = 25 μM, $[Na_2SO_3]_0$ = 50 mM, N_2-purged, and pH 12. The incident UV fluence rates range from 3 to 3.63×10^{-9} mol photons·cm^{-2}·s^{-1} at 222 nm and from 0.99 to 1.21×10^{-8} mol photons·cm^{-2}·s^{-1} at 254 nm.[2]

Calculation of quantum yield for nitrate degradation in ROC under UV-only conditions

The nitrate degradation rate due to direct photolysis in ROC was estimated following the approach described in Equation (S7), which can be rearranged to yield the apparent photolysis rate constant

$$k_P = k_a \times \Phi = \Phi \frac{I_{0\lambda}}{\ell}\left(1 - 10^{-(\alpha_\lambda + \varepsilon_\lambda C_i)\ell}\right)\frac{\varepsilon_\lambda}{(\alpha_\lambda + \varepsilon_\lambda C_i)} \quad \text{Eq. S22}$$

where k_p is the apparent photolysis rate constant (h^{-1}), k_a is the photon absorption rate constant (h^{-1}), Φ is the quantum yield, $I_{0\lambda}$ is the incident photon irradiance in mmol photons/cm^2/s, ℓ is the pathlength of light in the water in cm, α_λ is the decadic absorbance of the solution (excluding the chemical of interest i), C_i is the concentration of the chemical of interest i in mol/L, ε_λ is the molar absorptivity of the chemical of interest in M^{-1} cm^{-1}, and F_i is the fraction of light absorbed by the chemical of interest is given by $\varepsilon_\lambda C_i / (\varepsilon_\lambda C_i + \alpha_\lambda)$.

For nitrate decay in ROC, the following parameters were used: Φ = 0.35, $I_{0\lambda}$ = 4.7 E-6 mmol photons·cm^{-2}·s^{-1}, ℓ = 1.02 cm, ε_λ = 2540 M^{-1}·cm^{-1} at 222 nm (Table S23). In ROC, the initial nitrate concentration in ROC is 2.5 mM (Table S22) and the absorption coefficient (α_λ) was determined to be 62.8 (cm^{-1}), based on a measured absorbance of 0.519 cm^{-1} at 222 nm after 121-fold dilution of ROC (Table S21). Using these parameters, k_p for nitrate photolysis in ROC was calculated to be 0.217 h^{-1} using Eq. (S22). In comparison, the experimentally observed nitrate decay rate in ROC in the presence of sulfite was 1.05 h^{-1} (Figure S25). These results indicate that approximately 21% of the observed nitrate decay can be attributed to UV photolysis, while the remaining fraction (~79%) is attributed to reactions with hydrated electrons.

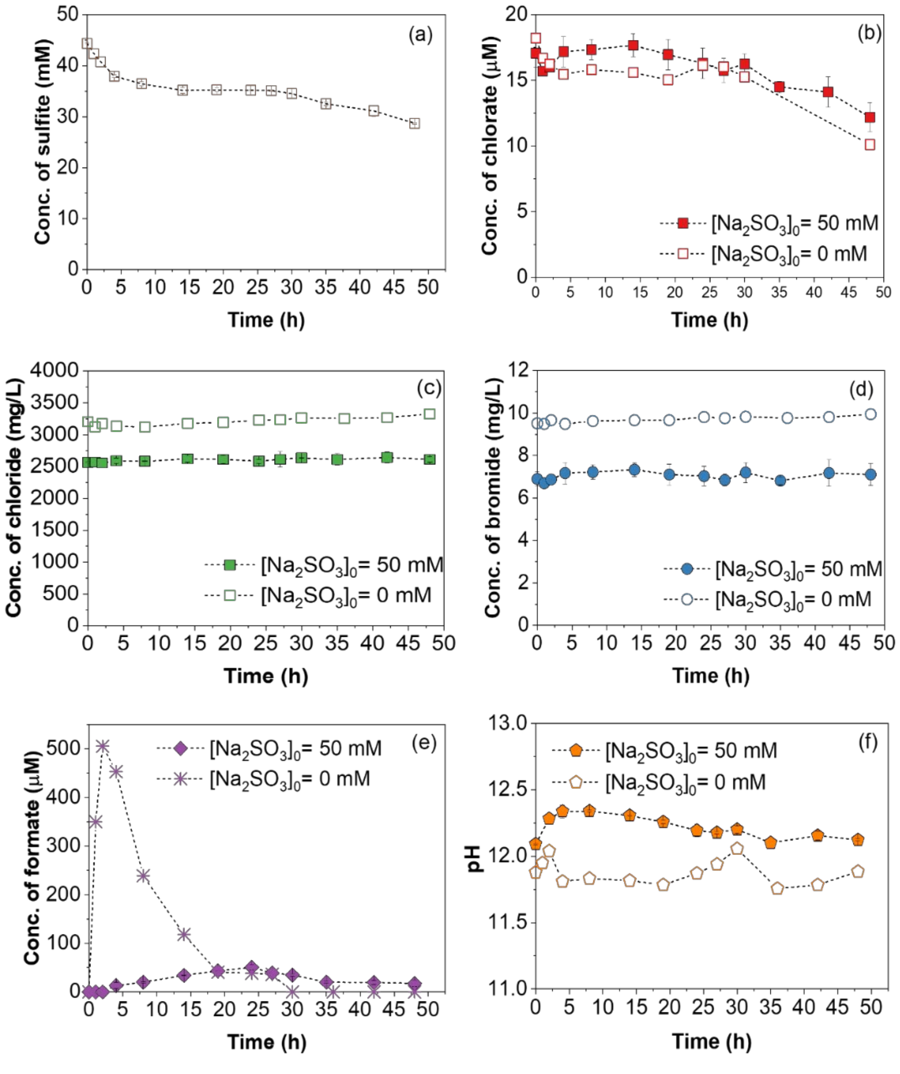

Figure S26. Time profiles of sulfite (a), chlorate (b), chloride (c), bromide (d), formate (e), and pH (f) during $PFAS_{mixture}$ decay in ROC by UV_{222} photolysis and UV_{222}/S-ARP. Conditions: $[PFAS_{mixture}]_0$ = 25 μM, N_2-purged, pH 12, and 222 nm irradiation (exp. 1-3 in Table S4). Marker represent the mean of duplicate measurements, and error bars indicate the range between duplicates.

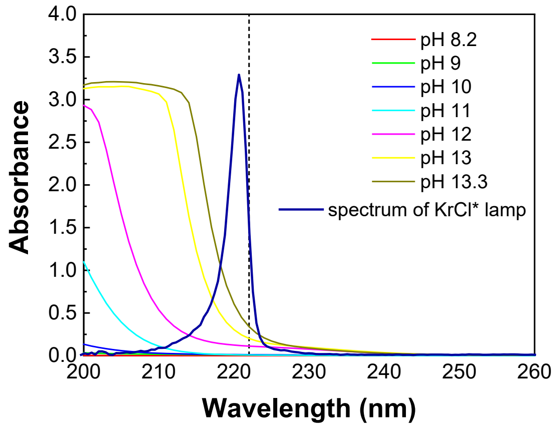

Figure S27. Absorption spectra of NaOH at different concentrations in aqueous solutions. Conditions: The solution concentrations were adjusted using deionized water.

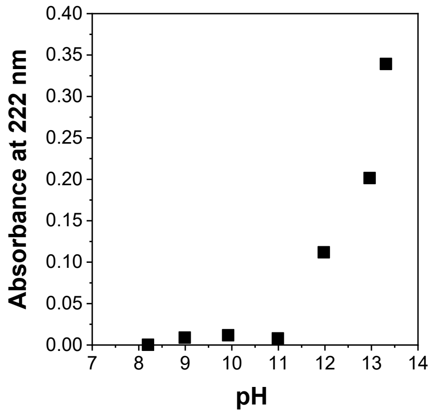

Figure S28. Absorbance of NaOH solutions at 222 nm as a function of pH.

Calculation of fluence-normalized deF of AFFF (1:60000 dilution) at 222 nm and 254 nm.

Table S25. Fluence-normalized defluorination of AFFF at 222 nm versus 254 nm.

Parameter	222 nm	254 nm[34]
AFFF dilution	1:60000	1:60000
$[Na_2SO_3]_0$	10 mM	10 mM
$[NaHCO_3]_0$ and pH	5 mM, pH 9[a]	5 mM, pH 9.5
N_2 saturation	Anaerobic	Anaerobic
Solution volume	~ 17 mL	~ 570 mL
Effective path length	1.02 cm	2.85 ± 0.03 cm
photon fluence rate	3.1E-9 ± 9E-11 mol photons·cm^{-2}·s^{-1}	1E-8 ± 3.3E-11 mol photons·cm^{-2}·s^{-1}
Photon energy	539 kJ·mol^{-1} per photon	472 kJ·mol^{-1} per photon
UV fluence rate	1.69 mW·cm^{-2}	4.71 mW·cm^{-2}
Defluorination % (deF)	86.2 % (48 h)	53% (49 h)
Fluence-normalized deF	0.296 %·cm^2·J^{-1}	0.064 %·cm^2·J^{-1}

[a]It is the measured pH at t=0.

References

1. Fuller, E. C.; Crist, R. H., The Rate of Oxidation of Sulfite Ions by Oxygen. *J. Am. Chem. Soc.* **1941,** *63*, (6), 1644–1650.

2. Fennell, B. D.; Chavez, S.; McKay, G., Destruction of Per- and Polyfluoroalkyl Substances in Reverse Osmosis Concentrate Using UV-Advanced Reduction Processes. *ACS EST Water* **2024,** *4*, (11), 4818–4827.

3. Cheng, L.-L.; Wang, M.; Zhu, H.; Li, K.; Zhu, R.-R.; Sun, X.-Y.; Yao, S.-d.; Wu, Q.-S.; Wang, S.-L., Characterization of the Transient Species Generated by the Photoionization of Berberine: A Laser Flash Photolysis Study. *Spectrochim. Acta A Mol. Biomol. Spectrosc.* **2009,** *73*, (5), 955 - 959.

4. Humphrey, R. E.; Ward, M. H.; Hinze, W., Spectrophotometric Determination of Sulfite with 4,4'-Dithiodipyridine and 5,5'-Dithiobis-(2-Nitrobenzoic Acid). *Analytical Chemistry* **1970,** *42*, (7), 698-702.

5. Fennell, B. D.; Odorisio, A.; McKay, G., Quantifying Hydrated Electron Transformation Kinetics in UV-Advanced Reduction Processes Using the R_{e-},UV Method. *Environ. Sci. Technol.* **2022,** *56*, (14), 10329-10338.

6. Yin, R.; Zhang, Y.; Zhao, J.; Shang, C.; Ren, H., Reductive Control of Refractory Contaminants in Water by Far-UVC Photolysis of Sulfite. *Environ. Sci. Technol.* **2025,** *59*, (27), 14161–14169.

7. Buxton, G. V.; Greenstock, C. L.; Helman, W. P.; Ross, A. B., Critical Review of Rate Constants for Reactions of Hydrated Electrons, Hydrogen Atoms and Hydroxyl Radicals (·OH/·O−) in Aqueous Solution. *J. Phys. Chem. Ref. Data* **1988,** *17*, (2), 513-886.

8. Butler, I. B.; Schoonen, M. A. A.; Rickard, D. T., Removal of dissolved oxygen from water: A comparison of four common techniques. *Talanta* **1994,** *41*, (2), 211-215.

9. Crittenden, J. C.; Hu, S.; Hand, D. W.; Green, S. A., A Kinetic Model for H_2O_2/UV Process In a Completely Mixed Batch Reactor. *Water Res.* **1999,** *33*, (10), 2315-2328.

10. Beltran, F. J.; Ovejero, G.; Garcia-Araya, J. F.; Rivas, J., Oxidation of Polynuclear Aromatic Hydrocarbons in Water. 2. UV Radiation and Ozonation in the Presence of UV Radiation. *Industrial & Engineering Chemistry Research* **1995,** *34*, (5), 1607-1615.

11. Xin, X.; Kim, J.; Ashley, D. C.; Huang, C.-H., Degradation and Defluorination of Per- and Polyfluoroalkyl Substances by Direct Photolysis at 222 nm. *ACS EST Water* **2023,** *3*, (8), 2776–2785.

12. Goss, K.-U., The pKa Values of PFOA and Other Highly Fluorinated Carboxylic Acids. *Environ. Sci. Technol.* **2008,** *42*, (2), 456–458.

13. Rayne, S.; Forest, K.; Friesen, K. J., Computational approaches may underestimate pKa values of longer-chain perfluorinated carboxylic acids: Implications for assessing environmental and biological effects. *Journal of Environmental Science and Health, Part A: Toxic/Hazardous Substances & Environmental Engineering* **2009,** *44 (4), 317-326*, (4), 317-326.

14. Rayne, S.; Forest, K.; Friesen, K., Extending the semi-empirical PM6 method for carbon oxyacid pKa prediction to sulfonic acids: Application towards congener-specific estimates for the environmentally and toxicologically relevant C1 through C8 perfluoroalkyl derivatives. *Nature Precedings* **2009**.

15. The Federal-Provincial-Territorial Committee on Drinking Water, Perfluorooctane Sulfonate (PFOS) in Drinking Water. In Health Canada: 2016.

16. Balasuryia, D.; Queral-Beltran, A.; Vick, T.; Simpson, S.; Lacorte, S.; Aga, D. S.; Hoepker, A. C., Experimental Determination of pKa for 10 PFAS, Mono-, Di-, and Trifluoroacetic Acid by 19F-NMR. *Environ. Sci. Technol. Lett.* **2025,** *12*, (9), 1238–1246.

17. Nong, Y.-J.; Wu, Q.-Y.; Qin, Y.-C.; Jing, Z.-B.; Wang, E.-D.; Bai, Q.; Wang, W.-L., Enhanced Photolysis of Sulfite by Far-UVC (222 nm) and Efficient Photoreductive Degradation and Dehalogenation of Halogenated Organic Pollutants. *Environ. Sci. Technol.* **2025,** *59*, (30), 16011–16020.

18. Dixit, F.; Barbeau, B.; Mostafavi, S. G.; Mohseni, M., Removal of Legacy PFAS and Other Fluorotelomers: Optimized Regeneration Strategies in DOM-rich Waters. *Water Research* **2020,** *183*.

19. Li, X.; Ma, J.; Liu, G.; Fang, J.; Yue, S.; Guan, Y.; Chen, L.; Liu, X., Efficient Reductive Dechlorination of Monochloroacetic Acid by Sulfite/UV Process. *Environ. Sci. Technol.* **2012,** *46*, (13), 7342−7349.

20. Shang, C.; Yin, R.; Zhang, Y., Rapid Dehalogenation of Refractory Organic Contaminants in Water by Far-UVC Photolysis of Sulfite. In Paper presented at 2023 IUVA World Congress: 2024.

21. Mack, J.; Bolton, J. R., Photochemistry of Nitrite and Nitrate in Aqueous Solution: A Review. *J. Photochem. Photobiol. A: Chemistry* **1999,** *128*, (1-3), 1-13.

22. Xu, J.; Huang, C.-H., Enhanced Direct Photolysis of Organic Micropollutants by Far-UVC Light at 222 nm from KrCl* Excilamps. *Environ. Sci. Technol. Lett.* **2023,** *10*, (6), 543-548.

23. Zhao, J.; Payne, E. M.; Liu, B.; Shang, C.; III, E. R. B.; Mitch, W. A.; Yin, R., Making waves: Opportunities and challenges of applying far-UVC radiation in controlling micropollutants in water. *Water Res.* **2023,** *241*.

24. Yu, K.; Li, X.; Chen, L.; Fang, J.; Chen, H.; Li, Q.; Chi, N.; Ma, J., Mechanism and Efficiency of Contaminant Reduction by Hydrated Electron in the Sulfite/Iodide/UV Process. *Water Res.* **2017,** *129*, 357-364.

25. Iwata, A.; Nakashima, N.; Kusaba, M.; Izawa, Y.; Yamanaka, C., Quantum Yields of Hydrated Electrons by UV Laser Irradiation *Chem. Phys. Lett.* **1993,** *207*, (2,3), 137-142.

26. Guo, K.; Wu, S.; Qin, W.; Xie, R.; Wu, Y.; Li, X.; Ouyang, G.; Fang, J., Overlooked Generation of Reactive Oxidative Species from Water and Dioxygen by Far UV Light. *Environ. Sci. Technol.* **2024,** *58*, (50), 22431–22441.

27. Saxton, B.; Langer, T. W., The Ionization Constant of Monochloroacetic Acid, at 25°, from Conductance Measurements1. *J. Am. Chem. Soc.* **1933,** *55*, (9), 3638–3645.

28. Vidyarthi, S. K. Flash photolysis of intermediates in hydrated electron reactions. University of British Columbia, 1973.

29. Lian, R.; Oulianov, D. A.; Crowell, R. A.; Shkrob, I. A., Electron Photodetachment from Aqueous Anions. 3. Dynamics of Geminate Pairs Derived from Photoexcitation of Mono- vs Polyatomic Anions. *J. Phys. Chem. A* **2006,** *110*, 9071-9078.

30. Sauer, M. C.; Crowell, R. A.; Shkrob, I. A., Electron Photodetachment from Aqueous Anions. 1. Quantum Yields for Generation of Hydrated Electron by 193 and 248 nm Laser Photoexcitation of Miscellaneous Inorganic Anions. *J. Phys. Chem. A* **2004,** *108*, (25), 5490–5502.

31. Cheng, F.; Wang, J., Regulating Hydroxyl Radicals (•OH) and Hydrated Electron (eaq) for Enhanced Radiolytic Degradation of 4-Nitrophenol by Addition of H2O2. *ACS EST Water* **2024,** *4*, (11), 5077–5088.

32. Xu, J.; Kann, R. J.; Mohammed, D.; Huang, C.-H., Far-UVC 222 nm Treatment: Effects of Nitrate/Nitrite on Disinfection Byproduct Formation Potential. *Environ. Sci. Technol.* **2024,** *58*, (34), 15311–15320.

33. Goldstein, S.; Rabani, J., Mechanism of Nitrite Formation by Nitrate Photolysis in Aqueous Solutions: The Role of Peroxynitrite, Nitrogen Dioxide, and Hydroxyl Radical. *J. Am. Chem. Soc.* **2007,** *129*, (34), 10597-10601.

34. Tenorio, R.; Liu, J.; Xiao, X.; Maizel, A.; Higgins, C. P.; Schaefer, C. E.; Strathmann, T. J., Destruction of Per- and Polyfluoroalkyl Substances (PFASs) in Aqueous Film-Forming Foam (AFFF) with UV-Sulfite Photoreductive Treatment. *Environ. Sci. Technol.* **2020,** *54*, (11), 6957-6967
